# A Clot Twist: Extreme Variation in Coagulotoxicity Mechanisms in Mexican Neotropical Rattlesnake Venoms

**DOI:** 10.3389/fimmu.2021.612846

**Published:** 2021-03-11

**Authors:** Lorenzo Seneci, Christina N. Zdenek, Abhinandan Chowdhury, Caroline F. B. Rodrigues, Edgar Neri-Castro, Melisa Bénard-Valle, Alejandro Alagón, Bryan G. Fry

**Affiliations:** ^1^Venom Evolution Lab, School of Biological Sciences, University of Queensland, St Lucia, QLD, Australia; ^2^Institute of Biology Leiden (IBL), Leiden University, Leiden, Netherlands; ^3^Department of Biochemistry and Microbiology, North South University, Dhaka, Bangladesh; ^4^Laboratório de Herpetologia, Instituto Butantan, São Paulo, Brazil; ^5^Instituto de Biotecnología, Universidad Autónoma de México, Cuernavaca, Mexico

**Keywords:** rattlesnakes, venom, Mexico, blood, coagulotoxicity, snakebite

## Abstract

Rattlesnakes are a diverse clade of pit vipers (snake family Viperidae, subfamily Crotalinae) that consists of numerous medically significant species. We used validated *in vitro* assays measuring venom-induced clotting time and strength of any clots formed in human plasma and fibrinogen to assess the coagulotoxic activity of the four medically relevant Mexican rattlesnake species *Crotalus culminatus, C. mictlantecuhtli, C. molossus*, and *C. tzabcan*. We report the first evidence of true procoagulant activity by Neotropical rattlesnake venom in *Crotalus culminatus*. This species presented a strong ontogenetic coagulotoxicity dichotomy: neonates were strongly procoagulant *via* Factor X activation, whereas adults were pseudo-procoagulant in that they converted fibrinogen into weak, unstable fibrin clots that rapidly broke down, thereby likely contributing to net anticoagulation through fibrinogen depletion. The other species did not activate clotting factors or display an ontogenetic dichotomy, but depleted fibrinogen levels by cleaving fibrinogen either in a destructive (non-clotting) manner or *via* a pseudo-procoagulant mechanism. We also assessed the neutralization of these venoms by available antivenom and enzyme-inhibitors to provide knowledge for the design of evidence-based treatment strategies for envenomated patients. One of the most frequently used Mexican antivenoms (Bioclon Antivipmyn®) failed to neutralize the potent procoagulant toxic action of neonate *C. culminatus* venom, highlighting limitations in snakebite treatment for this species. However, the metalloprotease inhibitor Prinomastat substantially thwarted the procoagulant venom activity, while 2,3-dimercapto-1-propanesulfonic acid (DMPS) was much less effective. These results confirm that venom-induced Factor X activation (a procoagulant action) is driven by metalloproteases, while also suggesting Prinomastat as a more promising potential adjunct treatment than DMPS for this species (with the caveat that *in vivo* studies are necessary to confirm this potential clinical use). Conversely, the serine protease inhibitor 4-(2-aminoethyl)benzenesulfonyl fluoride hydrochloride (AEBSF) inhibited the direct fibrinogen cleaving actions of *C. mictlantecuhtli* venom, thereby revealing that the pseudo-procoagulant action is driven by kallikrein-type serine proteases. Thus, this differential ontogenetic variation in coagulotoxicity patterns poses intriguing questions. Our results underscore the need for further research into Mexican rattlesnake venom activity, and also highlights potential limitations of current antivenom treatments.

## Introduction

Snakebite is a major global health crisis, with an estimated total of 94,000–138,000 fatalities and at least 400,000 cases of permanent disabilities per year. These numbers are well-recognized as gross-underestimates due to poor or non-existent epidemiological record keeping in the most affected regions (1, 2). At the root of such dismal statistics is a combination of factors such as rampant poverty, a lack of professional medical assistance in snakebite hotspots—leading to the time-wasting use of ineffective traditional “remedies”—and antivenoms which may be ineffective, inaccessible, or unaffordable (2–7).

Antivenom has long been a neglected or “orphan” drug due to the high costs of production, limited markets, and the fact that it is needed the most by those who can afford it the least (3, 5). The market limitations are due to venom being an extremely dynamic trait with extensive variations occurring between distantly related species, regional variations across the range of a widely distributed species, or even variations during the different life-stages of an individual snake. All these factors may dramatically limit the efficacy of an antivenom, thereby restricting the scope of its use (4, 8, 9).

Of particular concern for antivenom production and efficacy are wide-ranging, taxonomically complex clades such as rattlesnakes (genera *Crotalus* and *Sistrurus*), which are responsible for most snakebite envenoming cases in the United States (10–13) and a significant proportion throughout Latin America (10, 14, 15). Rattlesnakes are a highly diverse clade of pit vipers (Viperidae: Crotalinae) found throughout the Americas from southern Canada to northern Argentina (10). It is therefore unsurprising that they have received considerable research attention, ranking among the most studied snake clade worldwide for decades (16) and serving as model organisms for numerous works in several fields such as biogeography (17, 18), evolutionary biology (19, 20), and ethology (21). These snakes have adapted to a variety of ecosystems, from tallgrass prairies and deserts, through tropical and temperate forests, which resulted in great phenotypic and ecological diversity within the group (18, 22, 23).

**Figure 1:**
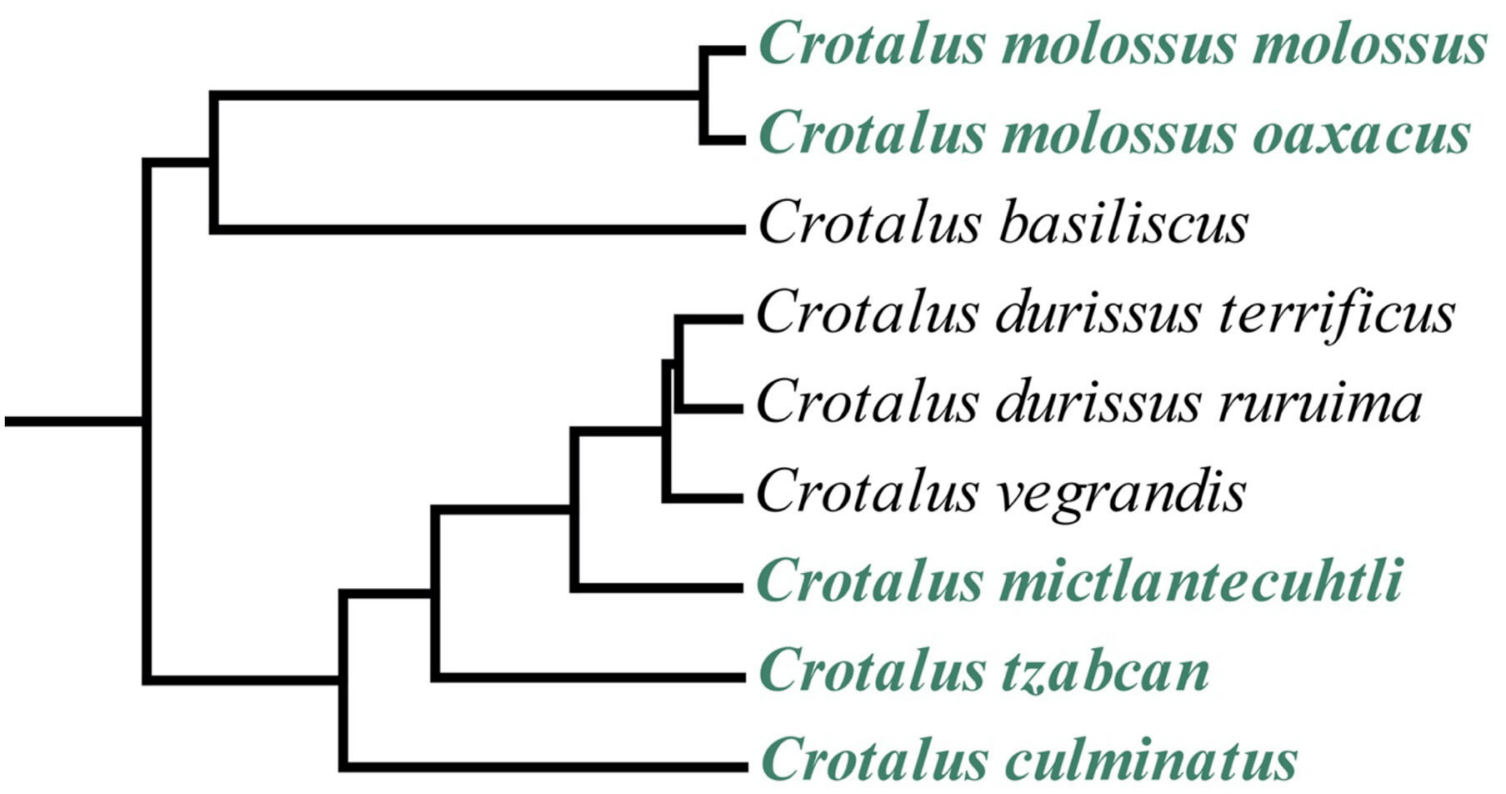
Phylogenetic tree of the *Crotalus durissus* group from a parallel study (timetree.org) showing the relationships between the *C. durissus* complex (Neotropical rattlesnakes) and the *C. molossus* complex. Species analyzed in this study are shown in green. Not all members of the clade are represented in the tree.

Mexico harbors the highest diversity of rattlesnake species in the world (22, 24). Among the most iconic and medically significant rattlesnake species in Mexico are the Neotropical rattlesnakes: *Crotalus culminatus, C. ehecatl, C. mictlantecuhtli, C. simus*, and *C. tzabcan*. These species are part of the *Crotalus durissus* complex, which also includes the eponymous species *C. durissus* alongside *C. vegrandis* (22, 25, 26) and is in turn included in the *C. durissus* group with a sister clade comprising *C. basiliscus, C. molossus, C. ornatus*, and *C. totonacus* [(22, 27), Figure 1]. These medium- to large-bodied rattlesnakes range from the southwestern United States (*C. molossus*) to northern Argentina (*C. durissus*), where they are responsible for a considerable number of serious envenoming cases (14, 15, 28–32).

A large body of research has been conducted on venom activity and composition in the *C. durissus* group. The widespread presence of the neurotoxic phospholipase A_2_ crotoxin in several species (33–37) places most Neotropical rattlesnakes (at least in early life stages) in the Type II venom category described by Mackessy (38). This class includes species possessing highly toxic venoms characterized by systemic neurotoxicity inducing rapid paralysis due to respiratory failure, rather than hemorrhagic symptoms (39–42). Conversely, phenotypes that are dominated by hemorrhagic and tissue-destroying snake venom metalloproteases (SVMPs) and generally devoid of neurotoxins are classified into the Type I category (38), which encompasses low-toxicity venoms inducing mostly cytotoxic and/or hemotoxic symptoms. However, the broad designation into Type I or Type II venoms does not fully account for factors such as ontogeny, prey specificity, intraspecific variation, and coagulotoxicity through differential biochemical pathways, and thus it is not reflective of actual biological diversity, which limits its categorical usefulness.

Neotropical rattlesnake venoms contain multiple toxins that disrupt hemostasis by targeting the blood clotting cascade, the concentration of which is often ontogenetic as well (35, 43–46). Research into coagulotoxicity produced by rattlesnake venoms has been largely focused upon anticoagulant toxins linked to the production of hemorrhagic shock through a combination of platelet inhibition, inhibition of activated clotting enzymes, depletion of fibrinogen levels, and degradation of the basement membrane of blood vessel walls leading to extravascular fluid loss (47–49). Fibrinogen depletion may occur in two ways, either *via* direct degradation by kallikrein-type serine proteases or metalloproteases, or through a pseudo-procoagulant action by 0005 kallikrein-type serine proteases where fibrinogen is converted to aberrant fibrin strands that form weak, transient clots that rapidly break down (50–54). Pseudo-procoagulant activity is distinguished from true procoagulant activity, i.e., the activation of clotting factors such as Factor X or prothrombin (55–59), by the nature of the fibrin clot formed. In pseudo-procoagulant venoms the direct action upon fibrinogen produces aberrant clots, while true procoagulant venoms generate endogenous thrombin which in turn produces well-ordered fibrin clots that contribute to the immobilization of prey through induction of stroke. In human victims, either scenario leads to venom-induced consumption coagulopathy (VICC), with extensive internal and external hemorrhage. Both SVMPs and snake venom serine proteases (SVSPs) are virtually ubiquitous across the rattlesnake clade, including Neotropical rattlesnakes (38).

In contrast to the well-documented anticoagulant effects, reports of true procoagulant activity in rattlesnake venom are scant and often inconclusive (60, 61), with the notable exception of *C. helleri* (62, 63). However, the paucity of data supporting the presence of true procoagulant toxins might have been influenced by intrinsic limitations in standard coagulotoxicity assays such as the procedure devised by Reid and Theakston (64), whereby Ca^2+^ and phospholipids are not added to citrated plasma prior to incubation with venom. As citration inactivates the clotting cascade by chelating ionized Ca^2+^, it is essential to add Ca^2+^ back in to reproduce physiological conditions. Furthermore, since plasma alone lacks both activated platelets and activated/apoptotic endothelial cells (i.e., the physiological source of phospholipids), its phospholipid concentration is likely low (65). Therefore, while trace amounts of phospholipids are present in citrated plasma, such small concentrations are not reflective of normal physiological conditions and would be rapidly depleted. Many studies have indeed clearly documented that both cofactors significantly affect relative coagulotoxicity (50–53, 56–59, 66–76). However, despite this critical importance having been known for decades, assay designs in many snake venom coagulotoxicity studies have included Ca^2+^ but not phospholipids (77–93) or neither of the clotting cofactors (32, 94–104). This may dramatically skew the results, to the point that procoagulant activity might be missed entirely for venoms that are inactive in the absence of clotting cofactors or generate enzymes such as FXa which are themselves obligately dependent upon Ca^2+^ for activity.

Since *in vitro* coagulotoxicity assays for the Mexican members of the *C. durissus* complex have largely followed methodologies that did not reproduce physiological conditions (32, 44), true procoagulant venom phenotypes could have gone undiscovered in this lineage. This could hamper antivenom efficacy and symptomatic treatment alike, as both anticoagulant and procoagulant venoms result in a net anticoagulant effect in human victims and thus cannot be distinguished on the basis of symptomatology. Therefore, in this study we investigated the clinical implications and possible evolutionary characteristics of coagulotoxicity in four species of the *C. durissus* group from Mexico, with a particular focus upon elucidating the type of coagulotoxicity (i.e., anticoagulant, pseudo-procoagulant, or true procoagulant) caused by the venoms. We assessed venom-induced clotting times and clot strength on human plasma and fibrinogen, ensuring to include Ca^2+^ and phospholipids in the assays to replicate physiological conditions, and testing clotting factor dependency under controlled conditions. We also tested the neutralization of these venoms by Bioclon Antivipmyn®, one of the main antivenoms marketed in Mexico, which is produced using *Bothrops asper* and *Crotalus simus* venom. We then repeated the tests using the commercially available metalloprotease inhibitors 2,3-dimercapto-1-propanesulfonic acid (DMPS) and Prinomastat, which have been shown to neutralize SVMPs in other venomous snake species (105, 106). Our findings provide valuable information for clinicians and antivenom producers regarding effective diagnosis and treatment of Neotropical rattlesnake envenoming in Mexico.

## Materials and Methods

### Venom Selection and Preparation

All venom work was performed under University of Queensland Approval #IBC134BSBS2015. Our study included 25 venom samples from *C. culminatus* (*n* = 15), *C. mictlantecuhtli* (*n* = 2), and *C. tzabcan* (*n* = 9), from the venom bank of the laboratory at IBt, UNAM (Herpetario Cantil). The *C. mictlantecuhtli* samples were obtained from pooling the venoms of juvenile (*n* = 5) and adult (*n* = 7) individuals. **Table 2** details the age category and locality of origin of each snake. Three venom samples from *C. molossus* (1 *C. m. molossus* and 2 *C. m. oaxacus*) were taken from the Venom Evolution Lab long-term cryogenic collection. *One* mg of each venom was transferred into a 1.5 mL Eppendorf tube under sterile conditions. Subsequently, ddH_2_O (double-distilled water) was added to the sample before vortexing for 5 s and centrifuging (4°C, 14,000 RCF; 10 min). The supernatant was then transferred to another 1.5 mL Eppendorf tube and the protein concentration determined in triplicate vortexing between replicates on a Nanodrop 2000 spectrophotometer at 280 nm (ThermoFisher Scientific). The resulting concentration values were used to obtain a final working stock of 1 mg/mL in 50% glycerol to prevent freezing at −20°C. Lastly, the samples were vortexed and aliquoted into 200 μL Eppendorf tubes for storage at −80°C until use. All venom samples were kept on ice throughout the process to avoid degradation.

### Plasma and Fibrinogen Coagulation Assays

All human plasma work was performed under University of Queensland Biosafety Approval #IBC134BSBS2015 and Human Ethics Approval #2016000256. Healthy human plasma (3.2%, citrated Lots# A540020142331 and # A5400201137021, which were pooled together) was provided by the Australian Red Cross (44 Musk Street, Kelvin Grove, Queensland 4059). Plasma stocks were aliquoted into 1.5 mL Eppendorf tubes under sterile conditions before flash-freezing in liquid nitrogen and stored at −80°C until use. Human fibrinogen was purchased from Sigma Aldrich (St. Louis, Missouri, United States, catalog #F3879) and aliquoted into 1.5 mL Eppendorf tubes after reconstitution into a running buffer (150 mM NaCl + 50 mM TrisHCl in 1 L ddH_2_O, pH 7.4) to a concentration of 4 mg/mL. The aliquots were flash-frozen in liquid nitrogen for 10 s and stored at −80°C until use.

Plasma and fibrinogen clotting times were measured on a Stago STA-R Max coagulation analyzer (Stago, Asniéres sur Seine, France) which determines clotting time *via* the time required for an oscillating magnetic ball inside a cuvette containing 250 μL solution to cease moving due to blockage caused by a clot. A detailed overview of the assays we performed is provided in Table 1. Prior to experimentation, a positive control for plasma was performed *via* an activated Partial Thromboplastin Time (aPTT) test as described by Lister et al. (107). A custom positive control assay was devised for fibrinogen whereby 50 μL 50% ddH_2_O:glycerol, 25 μL of a 2:1 dilution of CaCl_2_ + OK buffer, 50 μL phospholipids, and 75 μL human fibrinogen were incubated for 120 s before adding 50 μL thrombin (STA Liquid-FIB, Stago catalog # 00673) for a total volume of 250 μL. Negative controls for both plasma and fibrinogen were run by replacing the venom dilution with 50 μL 50% ddH_2_O:glycerol.

**Table 1:** Overview of coagulation assays performed on STA-R Max hemostasis analyzer.

**Assay**	**Methodology**
Venom-induced clotting time	**Step 1:** 50 μL venom (100 μg/mL) + 50 μL 0.025 M calcium (Stago catalog #00367) + 25 μL Owren-Koller (OK) buffer (Stago catalog #00360) + 50 μL phospholipids (Stago kit; catalog #00597)
	**Step 2:** 120 s incubation at 37°C + 75 μL human plasma/human fibrinogen
Calcium dependence	**Step 1**: 50 μL venom (100 μg/mL) + 75 μL OK buffer + 50 μL phospholipids
	**Step 2:** 120 s incubation at 37°C + 75 μL human plasma/human fibrinogen
Phospholipids dependence	**Step 1:** 50 μL venom (100 μg/mL) + 50 μL 0.025 M calcium + 75 μL OK buffer
	**Step 2:** 120 s incubation at 37°C + 75 μL human plasma/human fibrinogen
Antivenom	**Step 1:** 50 μL venom (100 μg/mL) + 50 μL 0.025 M calcium + 25 μL 2.5% antivenom + 50 μL phospholipids
	**Step 2:** 120 s incubation at 37°C + 75 μL human plasma/human fibrinogen
Prinomastat	**Step 1:** 50 μL venom (100 μg/mL) + 50 μL 0.025 M calcium + 25 μL 2 mM Prinomastat (Sigma-Aldrich, PZ0198-5MG) + 50 μL phospholipids
	**Step 2:** 120 s incubation at 37°C + 75 μL human plasma
DMPS	**Step 1:** 50 μL venom (100 μg/mL) + 50 μL 0.025 M calcium + 25 μL 2 mM/20 mM DMPS (ThermoFisher, U138044) + 50 μL phospholipids
	**Step 2:** 120 s/20 min incubation at 37°C + 75 μL human plasma
AEBSF	**Step 1:** 50 μL venom (100 μg/mL) + 50 μL 0.025 M calcium + 25 μL 2 mM AEBSF (Sigma-Aldrich, A8456-25MG) + 50 μL phospholipids

3.2% citrated plasma from cane toad (*Rhinella marina*) was aliquoted into 800 μL quantities, which were flash-frozen in liquid nitrogen, and stored at −80°C. This plasma was obtained under University of Queensland Animal Ethics Committee approval SBS/020/15/ARC.

### Cofactor Dependence Assays

To test whether the (pseudo)procoagulant action of venoms requires specific cofactors, dependence tests were performed using the plasma protocols from 2.2 on six representative Neotropical rattlesnake venoms (fastest- and slowest-clotting samples on plasma per species, with the exception of second-slowest adult *C. culminatus*) whereby the samples were incubated with human plasma and fibrinogen in the absence of either Ca^2+^ or phospholipids. Additional tests were conducted in a non-plasma assay which allowed for the strict control of either co-factor (see section Blood Clotting Factor Activation Assay below).

### Antivenom Neutralization and Inhibition Assays

One bottle of lyophilized Antivipmyn® antivenom serum [Instituto Bioclon, Calz. de Tlalpan 4691, Mexico City, Mexico; batch: B-6F-16, expiry date October 2010 and protein concentration of 13.7 mg *F*(ab′)_2_/mL] was diluted in 10 mL ddH_2_O and centrifuged (3,900 RCF, 4°C, 10 min) to remove any potential particulates. Expired antivenoms were not a concern, as antivenoms have been shown to be stable over time, with powdered antivenoms shown to be particularly resilient but even liquid antivenoms have been shown to be active for at least 60 years (107–109). Subsequently, the antivenom mixture was filtered (0.45 μm) and aliquoted into 2 mL Eppendorf tubes in sterile conditions, then stored at +4°C until use. For testing in STAR-Max, the antivenom was diluted in OK buffer to a 2.5% concentration, as determined to be effective during preliminary testing against *C*. *mictlantecuhtli* (formerly *C. simus* from Veracruz, Mexico) due to the presence of venom from this species in the immunizing mixture. Eight-point dilution curves were run for six venoms incubated at eight different concentrations (μg/mL: 20, 10, 4, 1.66, 0.66, 0.25, 0.125, and 0.05).

To test for inhibition of venom metalloprotease activity on plasma, eight-point curves were run on two representative venoms whereby the metalloprotease inhibitors Prinomastat hydrochloride (catalog #PZ0198, Sigma Aldrich, St. Louis, Missouri, US) and 2,3-dimercapto-1-propanesulfonic acid (DMPS, catalog #D8016 Sigma Aldrich, St. Louis, Missouri, US) replaced OK buffer as reagents in separate assays. Prinomastat was solubilized in DMSO, diluted to a 10 mM concentration using ddH_2_O, and subsequently stored at −80°C until use in STA-R Max. For this step, the inhibitor aliquots were thawed and pooled to a 900 μL total volume diluted into 3,600 μL OK buffer to dilute the concentration to 2 mM. DMPS was solubilized in DMSO and diluted in ddH_2_O to a 20 mM concentration before storage at −80°C. Prinomastat and DMPS aliquots were covered in aluminum foil to prevent exposure to light and degradation. Antivenom and inhibitor testing were performed using pooled plasma batch # A540020103540). We repeated the original baseline values for all species to demonstrate congruence and the plotting of dilution curves for the species upon which antivenom and inhibitors were tested.

Inhibition of serine protease activity on fibrinogen in a representative venom was assessed by running an eight-point curve with the serine protease inhibitor 4-(2-aminoethyl)benzenesulfonyl fluoride hydrochloride (AEBSF, catalog #A8456, Sigma Aldrich, St. Louis, Missouri, US) as a reagent in place of OK buffer. AEBSF was diluted with ddH_2_O into 20 mM aliquots which were covered in aluminum foil and stored at −80°C until use. For testing in STA-R Max, a 20 min incubation step with AEBSF was included before addition of fibrinogen as per (52).

### Thromboelastography

To assess the strength of venom-induced clots in plasma and fibrinogen, thromboelastography was performed on nine representative venoms using a Thromboelastogram® 5000 Hemostasis analyzer (Haemonetics®, Haemonetics Australia Pty Ltd., North Rdye, Sydney, Australia). The same ratio of reagents for STA-R Max assays was maintained for thromboestography. Briefly, 189 μL plasma (Label # A540020142331/A5400201137021) or fibrinogen (#Lot SLCC4502, #Lot SLBZ2294) were added to 72 μL CaCl_2_ (25 mM solution), 72 μL phospholipids diluted in OK buffer, 20 μL OK buffer, and 7 μL venom (1 mg/mL). Thromboelastography for *C. molossus* ssp. samples was performed using pooled plasma batch # A540020103540 due to degradation of the original plasma stock during a COVID-19 lockdown period, with the repeating of the original baseline values to demonstrate congruence. For plasma, a spontaneous (i.e., negative) clotting control was run with 50% ddH_2_O:glycerol in place of the venom, whereas 7 μL thrombin (STA Liquid FIB, Stago) or 7 μL bovine Factor Xa (Liquid Anti-Xa FXa, Stago) were used to run two independent positive controls. Only thrombin was used as a positive control for fibrinogen. Thromboelastography data were visualized on Adobe Photoshop.

### Blood Clotting Factor Activation Assay

Venom-induced activation of coagulation Factor II (prothrombin) and Factor X (FX) for nine representative venoms was investigated using a Fluoroskan™ microplate fluorometer (ThermoFisher Scientific, 168 Third Avenue, Waltham, MA 02451, USA). This machine measures activation of clotting factors by monitoring cleavage of a specific substrate (and corresponding fluorescence emitted) by an activated enzyme. The following reagents were manually pipetted into each experimental well in 384-well plates: 10 μL phospholipids (STA CK Prest, Stago), 10 μL venom, 10 μL zymogen. To determine the activity of the venom directly on the substrate, the zymogen was replaced with 10 μL Fluoroskan running buffer without Ca^2+^ (150 mM NaCl + 50 mM Tris, pH 7.4) in venom control wells. Activated factors replaced zymogens in positive control wells. A blank control without zymogen or venom was also included. ES011 substrate (Boc-Val-Pro-Arg-AMC. Boc: t-Butyloxycarbonyl; 7-Amino-4- methyl coumarin) was diluted to a 2 μg/mL concentration in Fluoroskan running buffer with 10 mM Ca^2+^ (150 mM NaCl + 50 mM Tris in 1 L ddH_2_O, + 10 mM Ca^2+^, pH 7.4). Seventy microliters of the dilution were then dispensed into each well by the machine to enable factor activation. All zymogens were diluted in Fluoroskan running buffer without Ca^2+^ to a 10 μg/mL concentration. Venom concentration was 1 μg/mL in running buffer without Ca^2+^ for FX activation. The prothrombin assay required the venom and zymogen concentrations to be lowered to 0.1 and 1 μg/mL, respectively, for subsequent analysis purposes due to the otherwise excessively high activity of the thrombin control. Activation was measured as the percentage of activated factor for each venom compared to the positive control (i.e., active enzyme wells), which represented the 100% activation benchmark. To test for cofactor dependence in FX activation, the assay was repeated by incubating venom with both cofactors vs. without phospholipids vs. without Ca^2+^.

### 1D Polyacrylamide Gel Electrophoresis (SDS-PAGE)

Non-reduced 1D 12% SDS-PAGE was run in triplicate to assess the activity of selected venoms on prothrombin. Venom (0.2 μg) was reconstituted in ddH_2_O and incubated at 37°C for 10 min with 2 μg prothrombin in a total volume of 7.5 μL. Negative (venom only; prothrombin only) and positive controls (thrombin only) were included in each gel. Then, 7.5 μL 2x laemmli dye (Bio-Rad Hercules, CA, USA) was added to each sample, resulting in a final volume of 15 μL. Lastly, the samples were stored at −20°C until use. Thirty milliliter of 12% resolving gel was prepared by pipetting 9.9 mL ddH_2_O, 12.0 mL 30% Acrylamide mix (Bio-Rad, Hercules, CA, USA), 7.5 mL 1.5 Tris-glycine pH 8.8 (Tris- Sigma Aldrich, St. Louis, MO, USA; glycine- Sigma Aldrich, St. Louis, MO, USA), 300 μL 10% SDS (SDS- Sigma-Aldrich, St. Louis, MO, USA), 300 μL 10% Ammoniun persulfate (APS- Bio-Rad, Hercules, CA, USA), and 18 μL TEMED in a 50 mL falcon tube. Six milliliter 5% stacking gel was prepared by pipetting 4.2 mL ddH_2_O, 990 μL 30% Acrylamide mix (Bio-Rad, Hercules, CA, USA), 750 μL 0.5 M Tris-glycine pH 6.8 (Tris- Sigma Aldrich, St. Louis, MO, USA; glycine- Sigma Aldrich, St. Louis, MO, USA), 60 μL 10% SDS (SDS- Sigma-Aldrich, St. Louis, MO, USA), 60 μL 10% APS (APS- Bio-Rad, Hercules, CA, USA), and 6 μL TEMED in a 15 mL falcon tube. Both gels were rested for 15 min to allow for polymerization before allocation into a Mini-PROTEAN Tetra Vertical Electrophoresis Cell (Bio-Rad, Hercules, CA, USA). 10x running buffer was prepared using the following recipe: 30 g Tris (Sigma Aldrich, St. Louis, MO, USA) + 144 g glycine (Sigma Aldrich, St. Louis, MO, USA) + 20 g SDS (Sigma Aldrich, St. Louis, MO, USA) diluted in 1 L ddH_2_O. Subsequently, 100 mL 10x running buffer was diluted in 900 mL ddH_2_O and poured into the electrophoresis chamber before manual loading of samples into the wells. A Dual Color protein standard (Bio-Rad, Hercules, CA, USA, range = 10–250 kD) was used as a ladder for molecular weight reference. Gels were run for 2.5 h at 120 V, then stained overnight with 1 g/L Coomassie colloidal brilliant blue G250 [34% methanol (VWR Chemicals, Tingalpa, QLD, Australia), 3% orthophosphoric acid (Merck, Darmstadt, Germany), 170 g/L ammonium sulfate (Bio-Rad, Hercules, CA, USA)] followed by destaining in ddH_2_O.

### Statistics

All tests were performed in quadruplicate (*n* = 4) bar the antivenom and inhibitor efficacy curves, which were run in triplicate (*n* = 3). Statistical analyses and graphing were performed in GraphPad PRISM v. 8.4.2. Cofactor dependence results were analyzed using repeated measures ANOVA *via* Dunnett's multiple comparisons test. This method allows for comparisons of each treatment (in our case, Ca^2+^-devoid and phospholipids-devoid conditions) to a control (normal conditions with both cofactors present). A repeated-measures approach was chosen because all clotting tests (i.e., control vs. treatment conditions) were conducted on the same venom sample for each species. Correlation tests were performed using Spearman's rank-order correlation due to age being coded as an ordinal variable with four categories (1 = neonate, 2 = juvenile, 3 = young adult, 4 = adult). Normality was determined with four different tests (Shapiro-Wilk, Kolmogorov-Smirnov, Anderson-Darling, D'Agostino and Pearson) but only the Shapiro-Wilk results were used since n = 4 was too small for the other tests. Significance was set at *p* = 0.05.

## Results

### Coagulotoxicity Assay

The venoms of neonate *C. culminatus* were strongly procoagulant, whereas adults appeared to have largely lost this trait (Table 2). Age of the animal and venom-induced clotting time were significantly correlated in *C. culminatus* for plasma (*r* = 0.8506, *p* < 0.0001) and fibrinogen (*r* = 0.7423, *p* < 0.0001). Both juvenile and adult *C. mictlantecuhtli* pools displayed short clotting times on plasma and especially fibrinogen, whereas greater individual variation was observed in *C. tzabcan* (Table 2).

**Table 2:** Clotting times of human plasma and fibrinogen incubated with venoms from *C. culminatus, C. mictlantecuhtli*, and *C. tzabcan* specimens.

**Species**	**Age**	**Locality**	**Mean clotting time (s) ± SD (plasma)**	**Mean clotting time (s) ± SD (fibrinogen)**
*C. culminatus*	Neonate	Tlaltizapán, Morelos	11.625 ± 0.33	31.775 ± 0.45
*C. culminatus*	Neonate	Yautepec, Morelos	14.975 ± 0.20	59.25 ± 3.10
*C. culminatus*	Neonate	Puente de Ixtla, Morelos	15.175 ± 0.35	51.3 ± 4.95
*C. culminatus*	Neonate	Iguala, Guerrero	40.25 ± 0.19	58.1 ± 1.84
*C. culminatus*	Juvenile	Coahuayana, Michoacán	14.4 ± 0.21	190.3 ± 14.57
*C. culminatus*	Juvenile	Coahuayana, Michoacán	16.925 ± 0.20	233.3 ± 3.81
*C. culminatus*	Juvenile	Morelos	18.075 ± 0.41	30.35 ± 1.03
*C. culminatus*	Juvenile	Morelos	85.525 ± 2.13	106.625 ± 0.45
*C. culminatus*	Juvenile	Barranca Honda, Morelos	107.65 ± 8.68	144.9 ± 2.95
*C. culminatus*	Young adult	Coahuayana, Michoacán	19.6 ± 0.33	108.775 ± 1.46
*C. culminatus*	Young adult	Puebla, Puebla	90 ± 1.78	128.5 ± 0.93
*C. culminatus*	Adult	Barranca Honda, Morelos	120.175 ± 6.09	192.075 ± 1.53
*C. culminatus*	Adult	Tlaltizapán, Morelos	122.725 ± 4.27	189.575 ± 6.02
*C. culminatus*	Adult	Barranca Honda, Morelos	181.175 ± 7.99	260.825 ± 20.39
*C. culminatus*	Adult	Cruz Pintada, Tlaltitenango, Morelos	218.75 ± 0.73	801.675 ± 65.32
*C. mictlantecuhtli*	Juvenile (pool *N* = 6)	Veracruz	46.275 ± 1.11	37.1 ± 0.46
*C. mictlantecuhtli*	Adult (poo *N* = 6l)	Veracruz	48.825 ± 0.52	41.775 ± 1.30
*C. tzabcan*	Neonate	Dzibilchatún, Yucatán	161.3 ± 4.65	149.65 ± 9.26
*C. tzabcan*	Juvenile	Calakmul, Campeche	79.425 ± 1.26	77.975 ± 2.24
*C. tzabcan*	Juvenile	Mérida, Yucatán	242 ± 57.00	265.6 ± 21.35
*C. tzabcan*	Juvenile	Chetumal, Quintana Roo	230.525 ± 1.80	325.45 ± 4.95
*C. tzabcan*	Adult	Solidaridad, Quintana Roo	73.225 ± 2.53	83.675 ± 4.01
*C. tzabcan*	Adult	Chetuma, Quintana Roo	112.975 ± 2.17	109.875 ± 6.95
*C. tzabcan*	Adult	Mérida, Yucatán	211.525 ± 7.31	223.875 ± 5.39
*C. tzabcan*	Adult	Oxkutzcab, Yucatán	267.55 ± 1.75	902.175 ± 119.35

*Plasma spontaneous control = 607 ± 23.39 s. Fibrinogen spontaneous control = 999 s*.

The cofactor dependence results confirm that coagulotoxins in the venom of these rattlesnakes are strongly dependent on cofactors, particularly Ca^2+^ (Tables 3, 4). Repeated-measures ANOVA yielded highly significant results regarding calcium dependence for all venoms on plasma, which was however markedly less pronounced for fibrinogen. Absence of phospholipids was not significant for one *C. tzabcan* sample and either *C. mictlantecuhtli* representatives (Table 3). Interestingly, all venoms clotted fibrinogen significantly faster in the absence of phospholipids than in normal conditions (Table 4). Relative co-factor dependence tests for zymogen activation by *C. culminatus* neonate were further investigated using completely controlled conditions in a non-plasma-based assay to eliminate the background fibrinogen-clotting effect (see section Blood Clotting Factor Activation Assay below).

**Table 3:** Cofactor dependence tests for six representative venoms (*C. culminatus, C. tzabcan*, and *C. mictlantecuhtli*) incubated with human plasma.

**Species**	**Locality**	**Normal**	**Phospholipid dependence (no phospholipids)**	**Ca^**2+**^ dependence (no Ca^**2+**^)**
*C. culminatus* neonate	Tlaltizapán, Morelos	11.63 ± 0.33	22.10 ± 0.46^***^	54.975 ± 0.80^***^
*C. culminatus* adult	Barranca Honda, Morelos	181.20 ± 7.99	178.62 ± 4.77	404.32 ± 17.65^**^
*C. tzabcan* adult	Solidaridad, Quintana Roo	73.23 ± 2.53	96.35 ± 3.16^***^	164.77 ± 2.62^***^
*C. tzabcan* adult	Oxkutzcab, Yucatán	267.6 ± 1.75	333.47 ± 21.88^*^	999 ± 0^***^
*C. mictlantecuhtli* juvenile (pool)	Veracruz	46.29 ± 1.14	48.025 ± 3.36	79.325 ± 0.80^***^
*C. mictlantecuhtli* adult (pool)	Veracruz	48.83 ± 0.52	53.8 ± 3.84	84.95 ± 1.25^***^

n = 4, values reported as mean ± SD. Statistical significance of cofactor-dependence tests compared to normal conditions (as inferred by Dunnett's multiple comparisons test following repeated-measures ANOVA) shown as follows:

* p < 0.05,

** p < 0.001,

*** p < 0.0001.

*All values represent venom-induced clotting times (s)*.

**Table 4:** Cofactor dependence tests for six representative venoms (*C. culminatus, C. tzabcan*, and *C. mictlantecuhtli*) incubated with human fibrinogen.

**Species**	**Locality**	**Normal**	**Phospholipid dependence (no phospholipids)**	**Ca^**2+**^ dependence (no Ca^**2+**^)**
*C. culminatus* neonate	Tlaltizapán, Morelos	33.75 ± 1.70	27.85 ± 0.17^*^	45.65 ± 0.34^*^
*C. culminatus* adult	Barranca Honda, Morelos	324.95 ± 15.73	190.20 ± 13.76^*^	764.60 ± 72.74^*^
*C. tzabcan* adult	Solidaridad, Quintana Roo	76.05 ± 2.88	39.43 ± 0.94^*^	120.50 ± 2.31^**^
*C. tzabcan* adult	Oxkutzcab, Yucatán	830.77 ± 52.91	333.47 ± 21.83^*^	999 ± 0^*^
*C. mictlantecuhtli* juvenile (pool)	Veracruz	34.88 ± 1.04	28.40 ± 0.33^*^	45.25 ± 0.64^*^
*C. mictlantecuhtli* adult (pool)	Veracruz	43.75 ± 1.60	33.25 ± 2.36^*^	61.75 ± 2.07^*^

n = 4, values reported as mean ± SD. Statistical significance of cofactor-dependence tests compared to normal conditions (as inferred by Dunnett's multiple comparisons test following repeated-measures ANOVA) shown as follows:

* p < 0.05,

** p < 0.001,

***p < 0.0001.

*All values represent venom-induced clotting times (s)*.

### Venom Neutralization by Antivenom and Inhibitors

**Figure 2:**
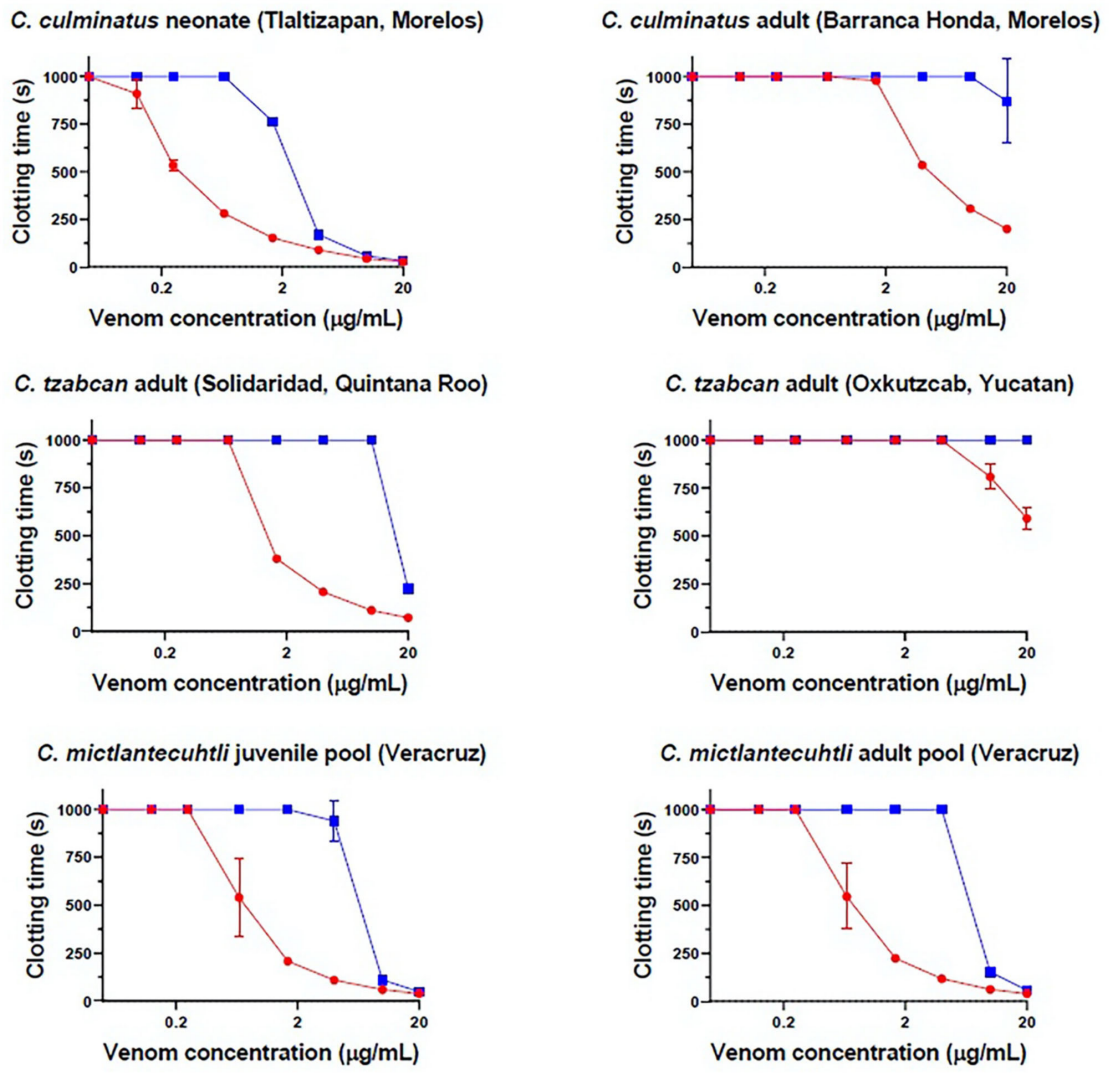
Eight-point dilution curves for six representative venoms against Antivipmyn® antivenom (2.5%) on human fibrinogen, Red = venom only, blue = venom + antivenom. X-axis is displayed in log form. Negative control = 999 + / − 0 s.

Eight-point dilution curves of antivenom efficacy indicate that Antivipmyn® effectively counteracts the pseudo-procoagulant action of neonate *C. culminatus, C. tzabcan* (Solidaridad), and both *C. mictlantecuhtli* pools, with a noticeable spike in antivenom efficacy from a 1.66 μg/mL venom dilution onwards (Figure 2). Venoms from adult *C. culminatus* and *C. tzabcan* (Oxkutzcab) only weakly affected fibrinogen compared to the other four samples, facilitating nearly complete neutralization of pseudo-procoagulant activity by the antivenom. *C. mictlantecuhtli* venom was also markedly neutralized by the serine protease inhibitor AEBSF (148 ± 5.62 s, *n* = 3, figure not shown).

**Figure 3:**
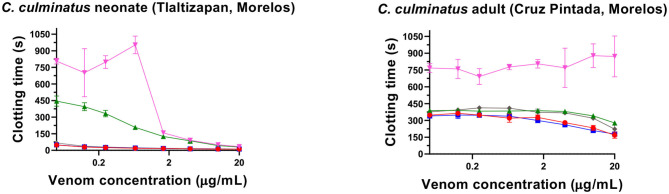
Eight-point dilution curves for *C. culminatus* venom upon plasma (red lines for venom-only) against Antivipmyn® antivenom (blue lines), 2 mM Prinomastat hydrochloride with 2 min incubation (green lines), 2 mM DMPS with 2 min incubation (pink lines). Spontaneous clotting control = 444.87 ± 26.73 s. Prinomastat negative control = 450.02 ± 57.33 s. DMPS negative control = 626 ± 36.23 s.

No detectable effect of Antivipmyn® was observed against neonate *C. culminatus* venom activity on plasma, and only marginal neutralization occurred against venom from an adult of the same species (Figure 2). Neonate *C. culminatus* venom-induced plasma clotting was instead greatly delayed by the metalloprotease inhibitor Prinomastat (particularly at low venom concentrations), whereas the adult individual was affected to a lesser degree. DMPS failed to neutralize either neonate or adult *C. culminatus* venom using the same assay as with Prinomastat (Figure 3). Furthermore, DMPS showed anticoagulant effects on plasma even in the absence of venom. Importantly, a different adult *C. culminatus* venom was used for antivenom + inhibitor tests and factor activation analysis (section Blood Clotting Factor Activation Assay) than the one used for clotting time assays and thromboelastography due to running out of the original stock. However, the results were congruent between the two venoms samples, which was consistent with both being from adult snakes from the same region.

### Thromboelastography

**Figure 4:**
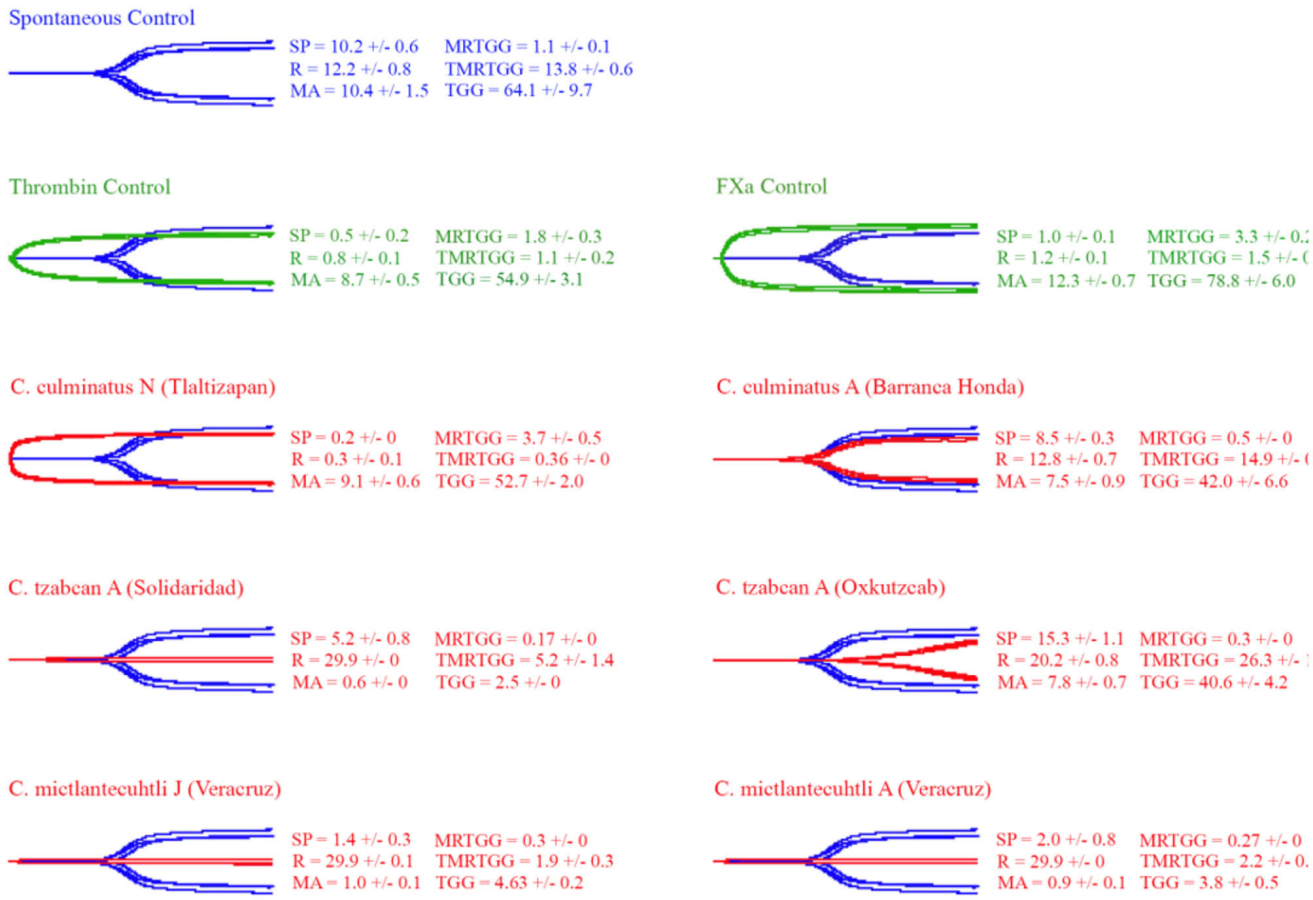
Thromboelastography traces showing the coagulation pattern of human plasma incubated with venoms of six Neotropical rattlesnake respresentatives for neonates (N), juveniles (J), and adults (A). Venom traces are shown in red, spontaneous clotting control traces in blue, thrombin and Fxa control traces in green. SP, split point (min); R, time (min) to minimum detectable clot (2 mm): MA, maximum amplitude (mm); MRTGG, maximum rate of thrombus generation (dynes/cm^2^); TMRTGG, time to maximum thrombus generation (min); TGG, total thrombus generation (dynes/cm^2^). Results shown as *n* = 4 mean ± SD.

**Figure 5:**
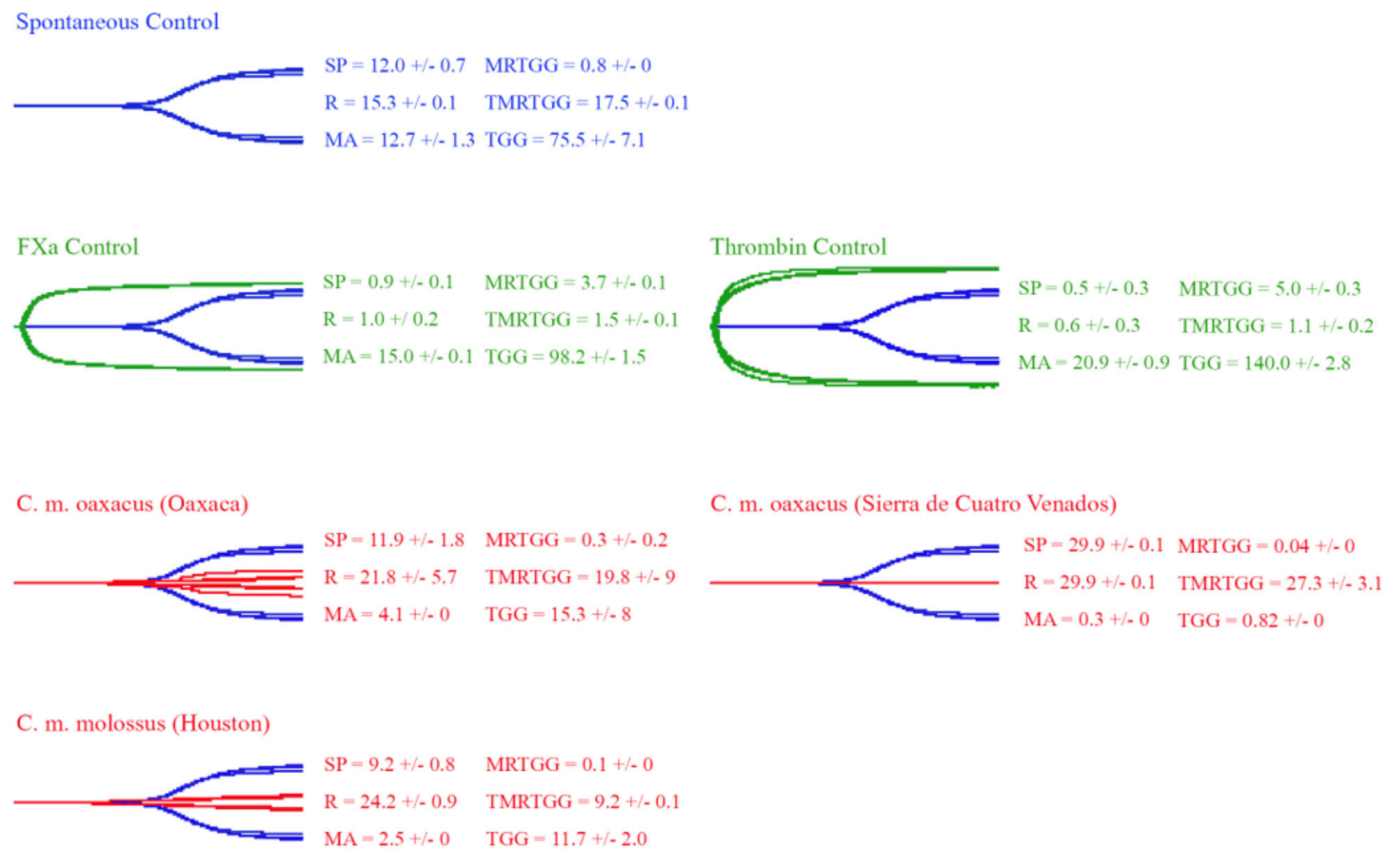
Thromboelastography traces showing the coagulation pattern of human plasma incubated with venoms of three *C. molossus* specimens of two subspecies (*C. m. molossus* and *C. m. oaxacus*). Venom traces are shown in red, spontaneous clotting control traces in blue, thrombin and Fxa control traces in green. SP, split point (min); R, time (min) to minimum detectable clot (2 mm); MA, maximum amplitude (mm); MRTGG, maximum rate of thrombus generation (dynes/cm^2^); TMRTGG, time to maximum thrombus generation (min); TGG, total thrombus generation (dynes/cm^2^). Results shown as *n* = 4 mean ± SD.

**Figure 6:**
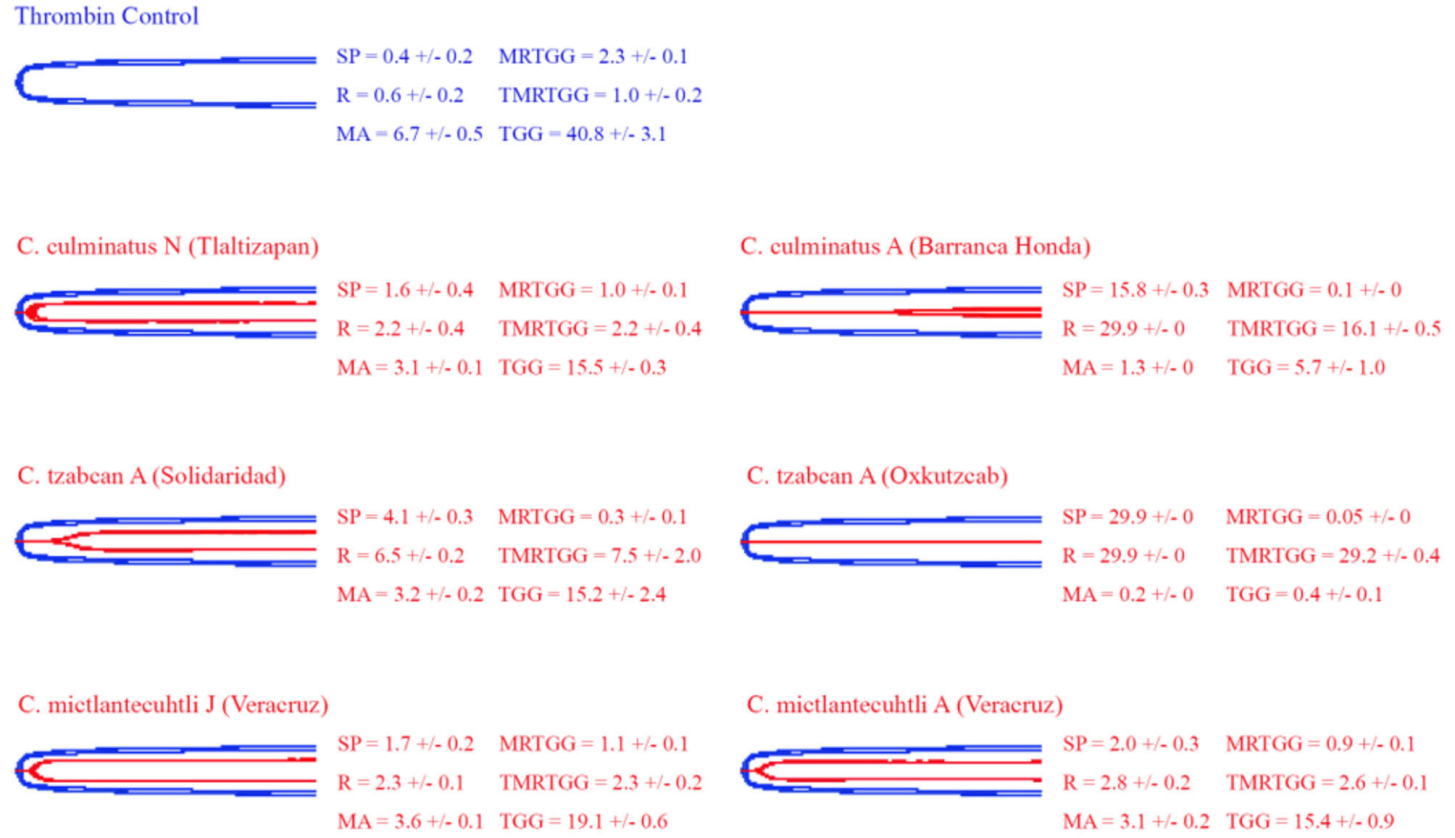
Thromboelastography traces showing the coagulation pattern of human fibrinogen incubated with venoms of six Neotropical rattlesnake respresentatives for neonates (N), juveniles (J), and adults (A). Venom traces are shown in red, thrombin control traces in blue. SP, split point (min); R, time (min) to minimum detectable clot (2 mm); MA, maximum amplitude (mm); MRTGG, maximum rate of thrombus generation (dynes/cm^2^); TMRTGG, time to maximum thrombus generation (min); TGG, total thrombus generation (dynes/cm^2^). Results shown as *n* = 4 mean ± SD.

**Figure 7:**
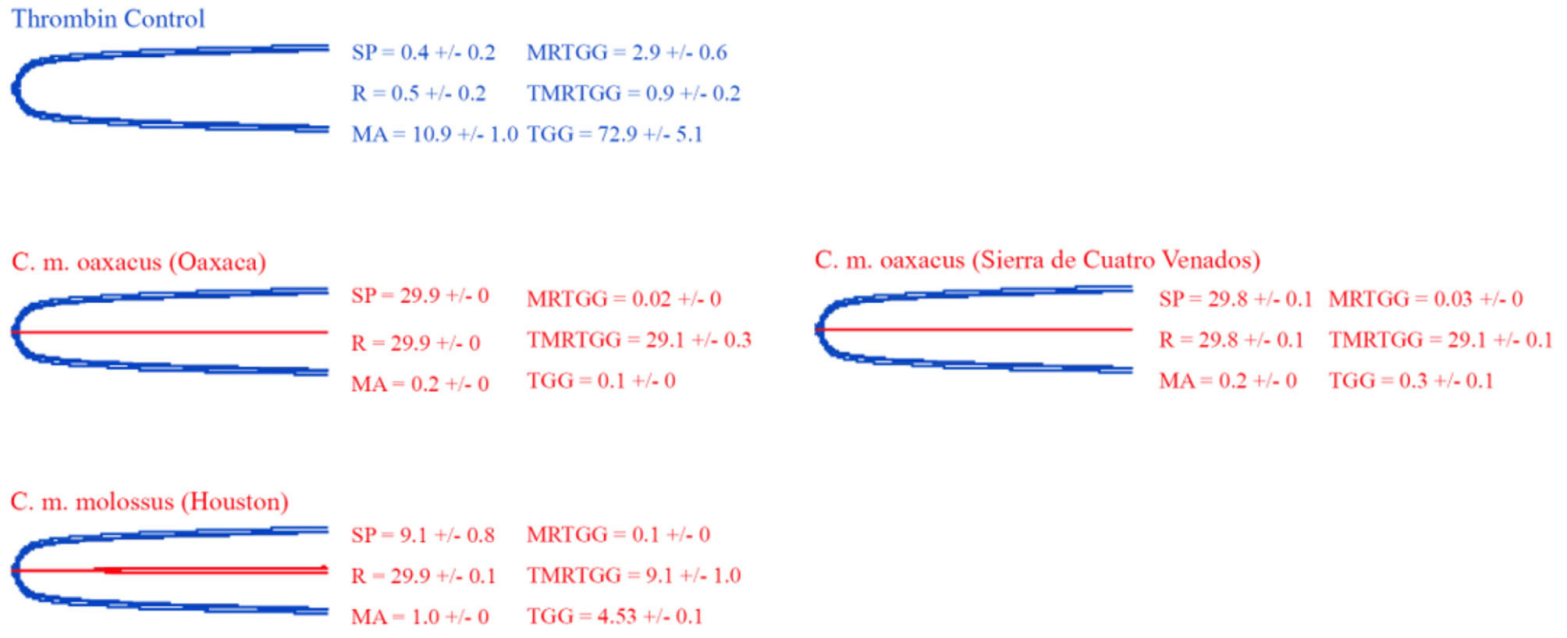
Thromboelastography traces showing the coagulation pattern of human fibrinogen incubated with venoms of three *C. molossus* specimens of two subspecies (*C. m. molossus* and *C. m. oaxacus*). Venom traces are shown in red, thrombin control traces in blue. SP, split point (min); R, time (min) to minimum detectable clot (2 mm); MA, maximum amplitude (mm); MRTGG, maximum rate of thrombus generation (dynes/cm^2^); TMRTGG, time to maximum thrombus generation (min); TGG, total thrombus generation (dynes/cm^2^). Results shown as *n* = 4 mean ± SD.

**Figure 8:**
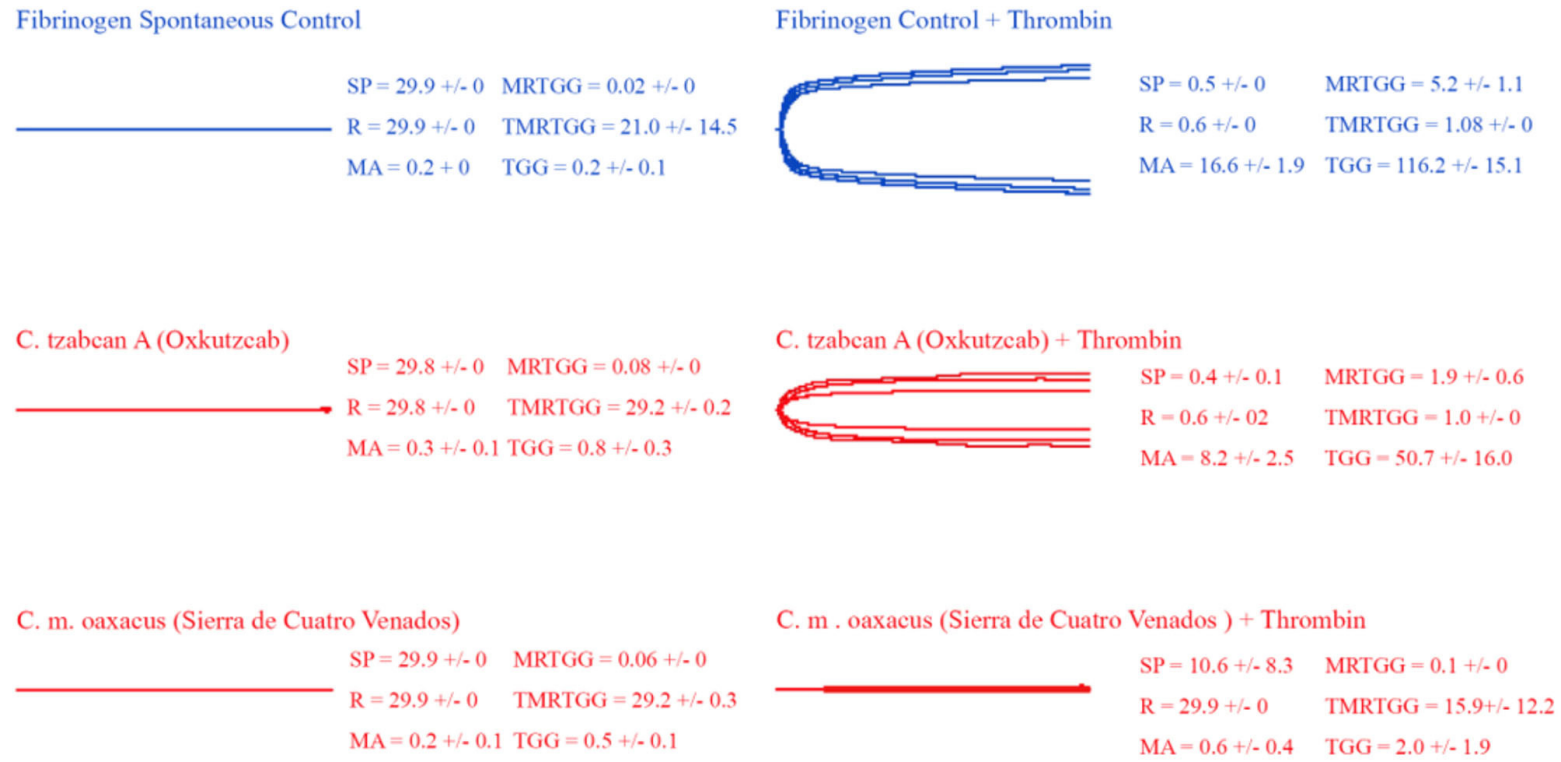
Thromboelastography traces showing the fibrinogenolytic action of venoms from *C. tzabcan* (Oxkutzcab) and *C. m. oaxacus* (Sierra de Cuatro Venados). Venom traces are shown in red, spontaneous and thrombin control traces in blue. SP, split point (min); R, time (min) to minimum detectable clot (2 mm); MA, maximum amplitude (mm); MRTGG, maximum rate of thrombus generation (dynes/cm^2^); TMRTGG, time to maximum thrombus generation (min); TGG, total thrombus generation (dynes/cm^2^). Results shown as *n* = 4 mean ± SD.

Thromboelastography was conducted on plasma and fibrinogen as follows: Figure 4 shows the human plasma thromboelastography traces for *C. culminatus, C. mictlantecuhtli*, and *C. tzabcan*. Figure 5 shows the human plasma thromboelastography traces for the three *C. molossus* localities; Figure 6 shows the human fibrinogen thromboelastography traces results for *C. culminatus, C. mictlantecuhtli*, and *C. tzabcan*; Figure 7 shows the human fibrinogen thromboelastography traces for the three *C. molossus* localities; Figure 8 shows the human fibrinogenolytic effects for *C. tzabcan* and *C. molossus oaxacus*.

Thromboelastography on plasma (Figures 4, 5) confirmed the marked procoagulant action of neonate *C. culminatus* venom on plasma on the STA-R Max assay, whereby a quick and strong clot was formed for the neonate, but not for the adult. The acceleration of clotting time and a strong, stable clot by the *C. culminatus* neonate venom is consistent with the activation of a clotting factor, which was specifically tested for in subsequent experiments (see section Blood Clotting Factor Activation Assay). None of the other venoms showed evidence of clotting factor activation in the plasma experiments (Figures 4, 5).

Thromboelastography on fibrinogen to test for pseudo-procoagulant fibrin-clot formation (Figures 6, 7) or destructive (non-clotting) fibrinogenolysis (Figure 8) also revealed sharp differences between age groups and species. The *C. culminatus* neonate retained as a background activity the basal pseudo-procoagulant activity widely present in rattlesnakes, but this trait was absent in the adult venoms. Intraspecific variation was evident in the *C. tzabcan* venoms, with one venom having pseudo-procoagulant activity upon fibrinogen whereas the other lacked this trait. Both neonate and adult *C. mictlantecuhtli* venoms displayed pseudo-procoagulant activity upon fibrinogen. This was not the case for *C. m. oaxacus*, while *C. m. molossus* showed only very slight activity in this regard. Further tests to see if *C. tzabcan* or *C. m. oaxacus* destructively cleaved fibrinogen revealed that while *C. tzabcan* did so only to a limited extent, *C. m. oaxacus* was extremely fibrinogenolytic, with the fibrinogen levels almost entirely depleted. Venoms were also tested on amphibian (cane toad) plasma, but none of them had any effect (data not shown).

### Blood Clotting Factor Activation Assay

**Figure 9:**
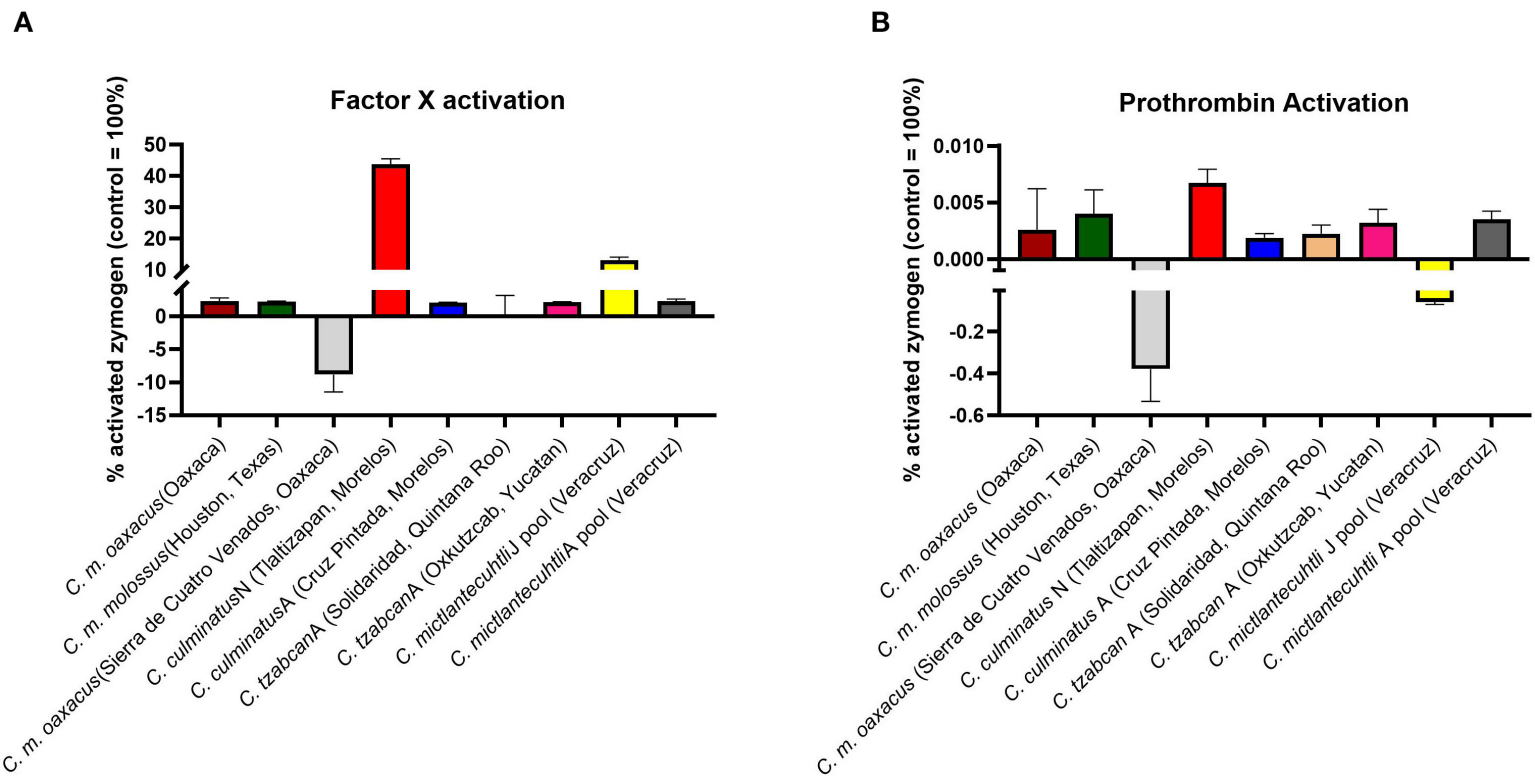
Fluorometry graphs showing activation of FX **(A)** and prothrombin **(B)** by selected rattlesnake venoms. Activation is expressed as the relative percentage of zymogen converted to its active form against a benchmark positive control incubated with thrombin and FXa, respectively (i.e., 100% active enzyme). Note the difference in Y-axis between FX activation and prothrombin activation, indicating considerably greater potency for the former. Values are *n* = 3 mean ± SD.

**Figure 10:**
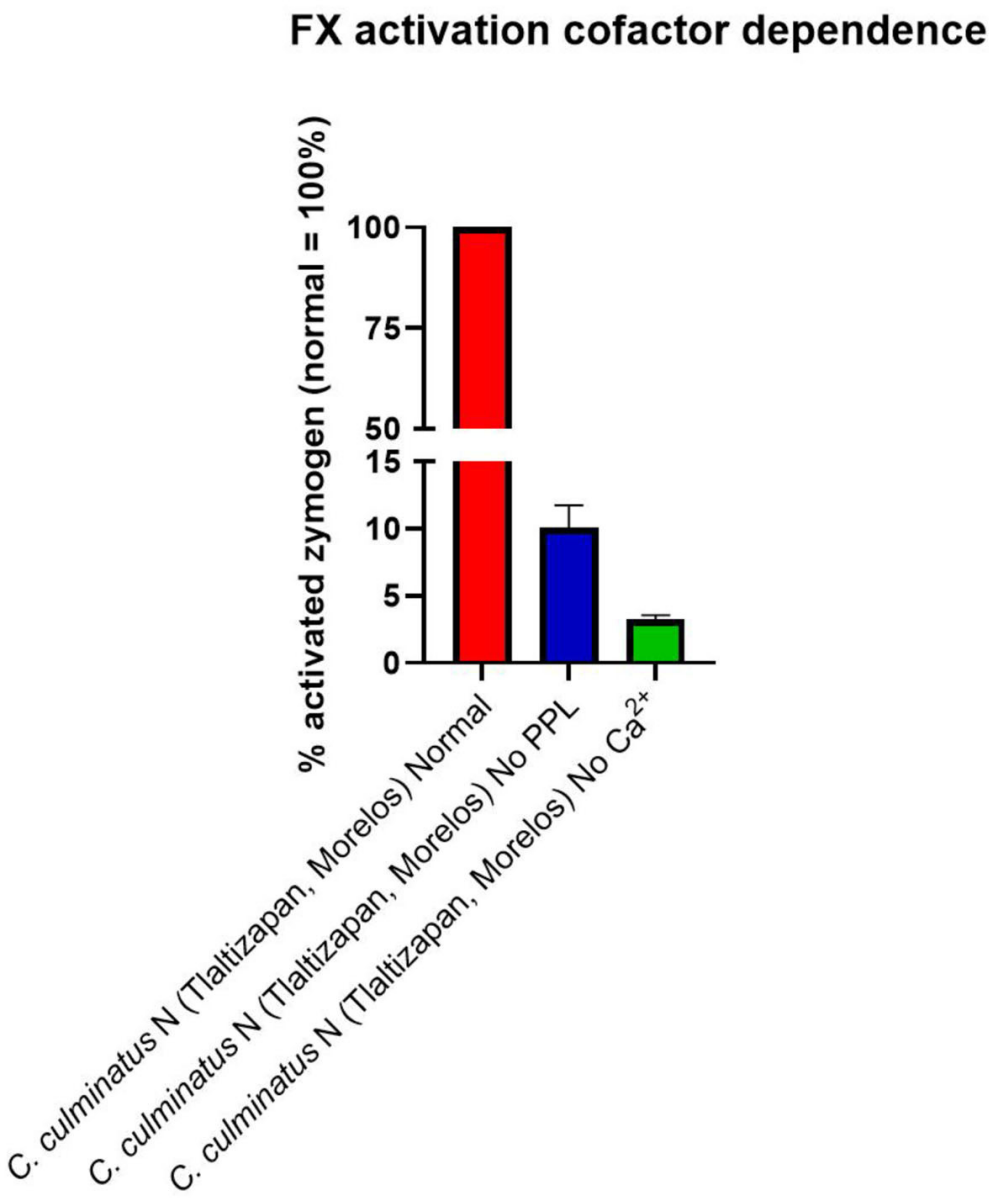
Fluorometry graph showing activation of FX by venom from a neonate *C. culminatus* specimen with and without coagulation cofactors (Ca^2+^ and phospholipids). Activation is expressed as the relative percentage of zymogen converted to its active form, with the normal condition (i.e., both Ca^2+^ and phospholipidss present in the incubation) as the 100% benchmark. Values are *n* = 3 mean ± SD.

As the prior results indicated that neonate *C. culminatus* venom was activating a clotting factor, tests were undertaken to test for activation of FII (prothrombin), FVII, FIX, FX, FXI, and XII. Only Factor X returned a strong result (Figure 9A), with prothrombin only being activated at a trace level (Figure 9B) and none of the other factors affected (data not shown). Consistent with the dichotomy observed on other clotting tests, the adult *C. culminatus* was 20-fold less potent than the neonate in the activation of FX and displayed no meaningful activity upon prothrombin or any other clotting factor. FX activation by neonate *C. culminatus* venom proved to be highly dependent on both calcium and phospholipids (Figure 10), the absence of which nearly abolished any action of the venom on the zymogen. Thus, the cofactor dependence values on whole plasma for this venom in Table 4 are artificially low due to the back-ground direct clotting of fibrinogen in a pseudo-procoagulant manner.

**Figure 11:**
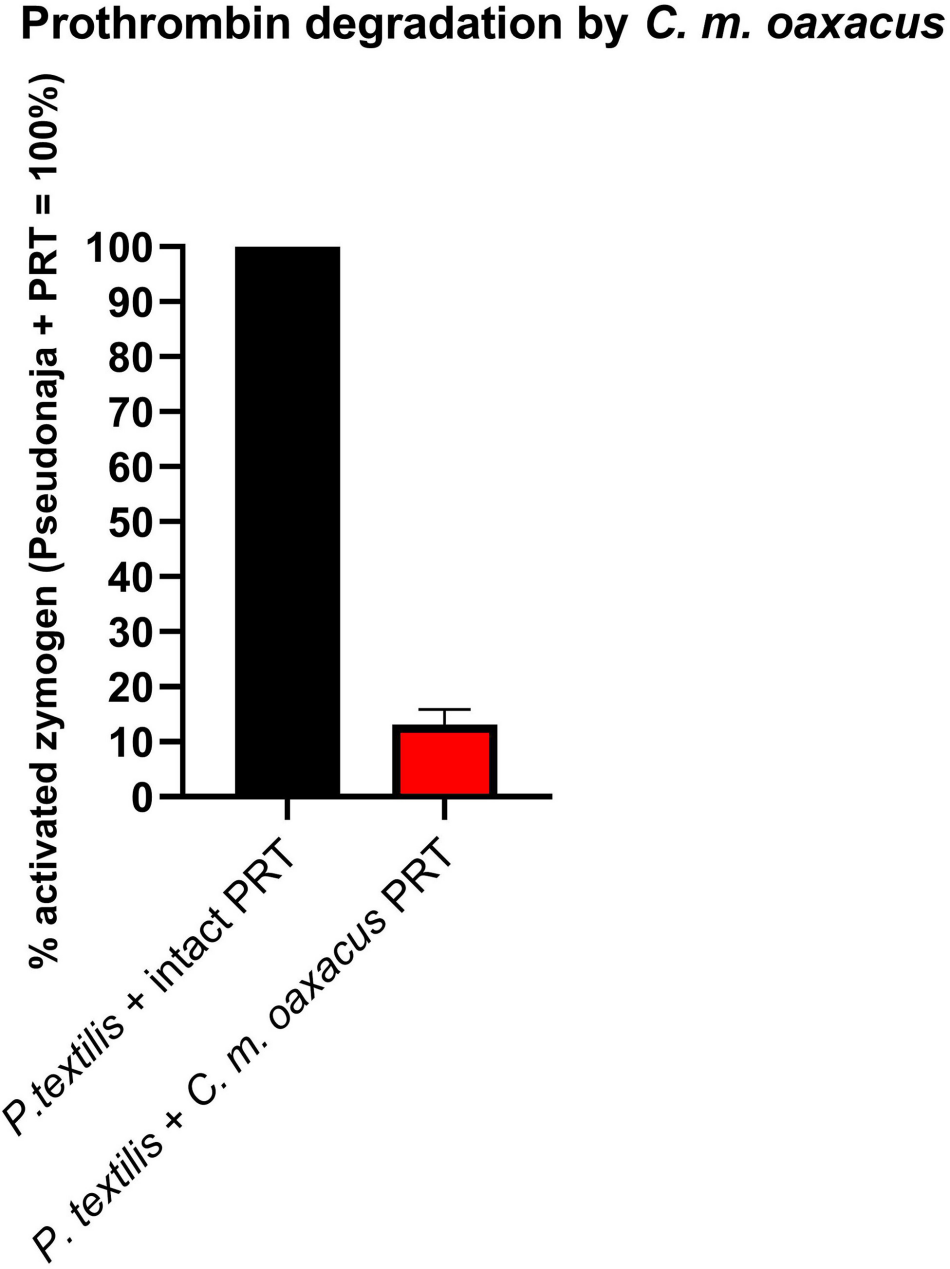
Fluorometry graph showing degradation of prothrombin (PRT) by venom from *C. m. oaxacus* (Sierra de Cuatro Venados). Degradation is expressed as the difference in *Pseudonaja*-activated zymogen levels between intact zymogen (black) and zymogen previously incubated with *C. m. oaxacus* venom (red). Values are *n* = 3 mean ± SD.

Intriguingly, *C. m. oaxacus* and *C. mictlantecuhtli* (juvenile pool) showed negative values. The [venom + substrate] controls were undertaken to provide the baseline activity of the venom in cleaving the substrate, with this amount to be subtracted from the results for the [venom + substrate + zymogen (FX or prothrombin)] experimental conditions. A negative value, whereby less fluorescence occurred for the [venom + substrate + zymogen] condition than for the [venom + substrate] indicates that in the [venom + substrate + zymogen] condition, less cleaving by the venom was observed than for the [venom + substrate] condition. This suggests that the venom was cleaving the substrate itself while simultaneously binding zymogen, resulting in less venom available to directly cleave the substrate when the zymogen was present. In addition, the interaction with the zymogen did not produce an active product from the cleaved zymogen. The ability to cleave the zymogen without yielding an active product could therefore represent a novel form of anticoagulation, as the zymogen would no longer be available to participate in the normal clotting cascade. This was evaluated experimentally with another fluorometric assay whereby *Pseudonaja textilis* venom, a well-known prothrombin activator (58), was incubated with intact zymogen and zymogen previously exposed to *C. m. oaxacus* venom for 1 h at 37°C. Activation in the *C. m. oaxacus*-treated zymogen was only 15% of that observed for the intact zymogen (Figure 11). Prothrombin degradation was further explored *via* gel electrophoresis [section 1D Polyacrylamide Gel Electrophoresis (SDS-PAGE)].

### 1D Polyacrylamide Gel Electrophoresis (SDS-PAGE)

**Figure 12:**
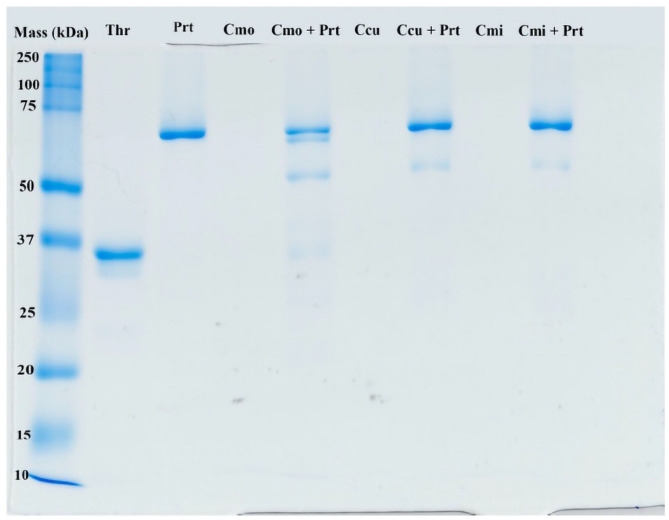
Representative ID gel of selected rattlesnake venoms incubated with prothrombin to illustrate prothrombin products. The ladder on the far left shows molecular weights (kDA = kilodaltons). Thr, thrombin (positive control, i.e., typical final product of prothrombin cleavage); Prt, prothrombin; Cmo, *C. m oaxacus*; Ccu, *C. culminatus*; Cmi, *C. mictlantecuhtli*.

1D SDS-PAGE of venoms incubated with prothrombin revealed clear differences in action of toxins from different species on this zymogen (Figure 12). In fact, *C. m. oaxacus* degraded prothrombin into several aberrant by-products spanning the region between the prothrombin and thrombin controls (72 and 36 kDa, respectively). On the other hand, *C. culminatus* (neonate) and *C. mictlantecuhtli* (pool of juveniles) affected the zymogen only weakly, with faint bands appearing in the 50–55 kDa region of the gel. A different neonate *C. culminatus* sample was used for this assay than in previous tests due to insufficient amount of venom remaining.

## Discussion

### Synopsis

This study aimed to shed light on the evolutionary history and medical consequences of coagulotoxicity in a group of Mexican rattlesnakes of high clinical concern and evolutionary novelty. To this end, we assessed coagulotoxic venom activities in these snake venoms *via* multiple different assays to produce a robust set of results. We reproduced physiological conditions as best as possible to accurately characterize the venom effects. In doing so we revealed a previously unknown ontogenetic variation in *C. culminatus*, whereby neonates are potently procoagulant through the activation of Factor X, but adults are pseudo-procoagulant in that they cleaved fibrinogen into unstable, short-lived fibrin clots, thus contributing to a net anticoagulant state by depleting fibrinogen levels. The *C. culminatus* FX activation was shown to be biochemically extremely reliant upon calcium and phospholipids. These results reinforce what a dynamic trait venom is, as the other species depleted fibrinogen levels either by pseudo-procoagulant actions on fibrinogen or through destructive (non-clotting) cleaving.

### Variations in Venom Biochemistry

Strikingly, we observed that venom from neonate *C. culminatus* clotted human plasma in our *in vitro* assay in 10–15 s, comparable to potently procoagulant snakes such as several Australian elapids (59, 76, 107, 110). We demonstrated that this activity was due to the activation of Factor X. The metalloprotease inhibitors Prinomastat and (to a much lesser degree) 2,3-dimercapto-1-propanesulfonic acid (DMPS) were effective in neutralizing the Factor X activation, revealing the activity to be driven by SVMP.

The unmistakably true procoagulant activity of *C. culminatus* venom was an unexpected finding in light of previous literature unanimously reporting a lack of any such trait in this species (32, 44). However, these studies did not include the clotting cofactors calcium or phospholipids in the assay conditions, which we show both clotting factors be critical through multiple assays in this study, and such cofactor dependence has long been documented in snake venoms (111). In addition to the venom activation of FX into FXa being obligately calcium-dependent, the bioactivity of the endogenous FXa which is produced by the venom is also obligately dependent upon calcium so even for venoms which are able to activate FX in the absence of calcium, their activity would be missed in assays which relied on protocol designs. The discrepancy between our results and previous literature is almost certainly due to the omission of clotting cofactors Ca^2+^ and phospholipids in prior research that relied upon the method developed in 1983 by Theakston and Reid (64), which did not include either clotting cofactor and has been largely followed with only minor modifications in toxicity studies of Mexican Neotropical rattlesnakes (32, 44, 112). Thus, calcium-obligate activities such as the Factor X activation discovered in this study would not be observable in assays lacking the clotting cofactors.

Such high levels of calcium dependence for procoagulant zymogen activation (Factor X or prothrombin) have been observed in other venomous snake lineages, including other pit vipers such as *Bothrops atrox* (113), true vipers of the genus *Echis* (56), Australian elapids (107) the genus *Atractaspis* within the Lamprophiidae family (57), and the colubrid genera *Dispholidus* and *Thelotornis* (74). In contrast, other genera are known to activate zymogens with much lower levels of calcium dependence, such as some species of *Echis* (56), and the Australian elapid genera *Oxyuranus* and *Pseudonaja* (58, 59). Venom-induced FX activation by neonate *C. culminatus* was also highly dependent on phospholipids, which appear to be nearly as crucial as Ca^2+^. This further highlights the importance for venom coagulotoxicity assays *in vitro* to include both cofactors so as to avoid skewing results.

The SVMP toxin class has been previously shown to be responsible for Factor X activation in a wide range of snakes, including the related pit viper genus *Bothrops* (113) and true vipers such as *Bitis worthingtoni* (76, 114). Thus, this trait either represents a remarkable case of functional convergence in the neofunctionalisation of an ancestral tissue-destroying metalloprotease or indicates that FX activation is an ancient trait that has been amplified on multiple convergent occasions but is only maintained at trace levels in most species. The answer to this question would require sequencing of the enzyme responsible for FX activation and reconstructing its molecular evolutionary history through the construction of a robustly supported molecular phylogenetic tree.

From a phylogenetic point of view, *C. culminatus* is consistently retrieved as an early divergence from the rest of the *C. durissus* complex (18, 26). Therefore, the true procoagulant venom phenotype observed in this species might have evolved independently or represented the ancestral state for this clade. The latter possibility was investigated by testing the venom of *C. molossus*, part of the sister clade to the *C. durissus* complex alongside *C. basiliscus, C. ornatus*, and *C. totonacus* (26, 27). However, our thromboelastography and factor activation results revealed only a weakly pseudo-procoagulant venom action for the nominate subspecies *C. m. molossus* and distinctly anticoagulant patterns for a *C. m. oaxacus* representative, which greatly degraded fibrinogen to a point where addition of thrombin was unable to form a clot. This is consistent with previous studies reporting high fibrin(ogen)olysis across the three subspecies of *C. molossus* (95, 115, 116) and does not support a procoagulant ancestral condition for the *C. durissus* group. Thus, this trait likely stems either from convergent amplifications of a basal FX-activating SVMP or convergent evolutions of neofunctionalised SVMPs in Viperidae.

The ability of the serine-protease inhibitor AEBSF to neutralize the pseudo-procoagulant activity of *C. mictlantecuhtli* venom demonstrated that this venom activity is driven by kallikrein-type serine proteases. The differential reliance upon Ca^2+^ extended to the pseudo-procoagulant actions on fibrinogen, with all the venoms acting notably more slowly (up to half as fast) in the absence of Ca^2^ (Table 4). The relative reliance upon phospholipids has also been shown to be a highly labile trait (66–73, 117). While the effect is less pronounced than for Ca^2+^, it is still a significant variable, showing extreme variation within a genus or even within different geographic ranges of a single species (50–53, 56–59, 107). Notably, our cofactor dependence assay revealed a consistently significant acceleration of fibrinogen clotting in the absence of phospholipids. This phenomenon was already observed in several Asian pitvipers of the genus *Trimeresurus* (52) and in the Australian elapid genus *Pseudonaja* (58). The biochemical dynamics underlying this pattern are unclear and warrant further research.

Another novel activity documented in this study was degradation of prothrombin by *C. m. oaxacus*, a phenomenon previously reported in several viper species (118, 119) but rarely in rattlesnakes (120). Such an activity would create a net anticoagulant state by depleting the amount of this endogenous clotting factor available for participation in the clotting cascade. This activity was first inferred from the negative values obtained in prothrombin activation tests, and then confirmed by two additional assays: first by incubating the venom with prothrombin, then adding a known prothrombin trigger, and comparing the results to the same trigger added to prothrombin that had not been exposed to *C. mo. oaxacus* venom; and secondly by an SDS-PAGE assay, whereby *C. m. oaxacus* produced several aberrant degradation by-products of higher molecular mass than thrombin. The net decrease in activity in the FX zymogen activation studies for *C. m oaxacus* is consistent with this species also degrading FX in addition to prothrombin. However, Factor X degradation was unable to be further examined due to running out of venom supplies. The fact that the venom produced the same negative values in the Flouroskan tests as was the case for prothrombin and with these negative values for prothrombin being confirmed by additional tests as indeed being reflective of degradation events, this is strongly suggestive of Factor X also being degraded by this venom. Future work to confirm this would involve assays such as were undertaken for prothrombin degradation in this study: (a) incubating the venom with Factor X, then adding a known Factor X trigger, and comparing the results to the same trigger added to Factor X that had not been exposed to *C. mo. oaxacus* venom; and (b) SDS-PAGE gels to ascertain relative cleavage products to determine if aberrant cleavage products were formed.

As discussed earlier, such stark individual variations are commonplace among rattlesnakes. *C. molossus* occurs throughout a vast range spanning from the southwestern US to southern Mexico, with blurred geographic and genetic boundaries among subspecies (10, 27). Our small sample size does not allow for documentation of subspecies- and population-level venom variability in this species, which therefore should be the subject of future research in order to elucidate to what extent venom variation reflects biogeographical and/or ecological drivers in the *C. molossus* complex.

### Prey-Capture Evolutionary Implications

The procoagulant activation of zymogens into their active forms (e.g., FX into FXa; prothrombin into thrombin) in prey animals would result in rapid incapacitation due to stroke, induced by large blood clots. Interestingly, procoagulant venom activity *via* FX activation in *C. culminatus* appears to be an ontogenetic trait, with the shortest and longest clotting times for both plasma and fibrinogen observed in neonates and adults, respectively. This is corroborated by our thromboelastography and fluorometry results in terms of time to clot formation and FX zymogen activation. Ontogenetic shifts in venom composition and/or activity have been extensively documented in a variety of rattlesnake species and lineages (60, 112, 121–124), particularly with respect to a pattern of loss of crotoxin-like neurotoxic PLA_2_s (Type II phenotype) in favor of hemorrhagic SVMPs (Type I phenotype) as the snake ages (38, 45, 46). This phenomenon is recurrent in the *C. durissus* complex (35, 43, 45). Such age-driven changes in venom composition are generally thought to stem from shifts in prey preference between juvenile and adult snakes (10, 121, 122), as seen in a variety of snakes ranging from Australian elapids (110) to lancehead pit vipers of the genus *Bothrops* (113, 125). However, our current knowledge—albeit fragmentary—points to *C. durissus, C. simus*, and *C. tzabcan* being rodent specialists throughout their life (10, 24, 126–129). While only scarce information is available for *C. culminatus*, reports indicate a rodent-centered diet as well (10, 23, 24, 130). This is supported by our thromboelastography results showing a strikingly potent procoagulant effect of *C. culminatus* venom on human plasma as opposed to no apparent activity at all on amphibian plasma, suggesting specialization for an endotherm-based diet. By contrast, the venoms of other vipers such as *Bitis worthingtoni* and several *Bothrops* representatives are known to activate both mammalian and amphibian plasma, with potency showing a clear correlation with degree of specialization on amphibian prey (113, 114). The Factor X zymogen differs significantly in mammals compared to amphibians and diapsids (i.e., reptiles and birds). Future work should investigate the lineage-specific motifs that guide such differential activation. Testing of *C. culminatus* venom on reptile plasma (e.g., lizard) would be a logical follow-up to corroborate our findings, since this snake occurs in dry habitats at mid- to high elevations in southwestern Mexico where other reptiles abound (23, 130, 131).

It has been suggested that potent, fast-acting toxins possibly serve as a means for small-sized snakes to rapidly incapacitate prey using a substantially lower amount of venom than adults are able to inject (122, 126) and/or to quickly immobilize prey items (113, 122, 126). Intriguingly, while nearly all members of the *C. durissus* complex present variable quantities of crotoxin in their venom, *C. culminatus* lacks this neurotoxin entirely (35, 45). By contrast, this species possesses a significantly higher percentage of SVMPs than *C. tzabcan* and *C. simus*, with neonates and juveniles possessing metalloproteases not found in adults and vice versa (44, 45). It is therefore possible that highly procoagulant SVMPs in early-stage *C. culminatus* play a role akin to that of crotoxin-like neurotoxins in other members of the *C. durissus* complex and other rattlesnake lineages, as factor-activating SVMPs are known to induce rapid death by stroke in small-sized animals (132). Neonate and juvenile rattlesnakes require meals as early as possible to avoid starvation and support high rates of growth (10, 133). Thus, a highly potent toxic component in neonate rattlesnake venom may greatly improve prey-capture and survival into adulthood. Our results align with the observations of Margres et al. (134) in the equally non-neurotoxic species *C. adamanteus*, with higher venom toxicity in juveniles compared to adults. This indicates that such a pattern may be widespread among rattlesnakes beyond the simplistic Type I vs. Type II categorization, an intriguing possibility that invites further research.

While *C. culminatus* possesses a distinctly Type I venom phenotype (44, 45), the SVMP-driven procoagulant activity observed in this study might serve a functional role analogous to that of neurotoxic PLA_2_ components in Type II venoms from juveniles of other species. Hence, a general classification such as the Type I vs. Type II dichotomy devised by Mackessy (38) might overlook peculiar toxic activities of venom in certain species and is therefore not reflective of the greater complexity present in biological reality. It must be noted, however, that SVMPs are considerably larger than crotoxin isoforms in terms of molecular weight, which might delay absorption *via* the bloodstream and/or lymphatic system, as documented for *C. simus* venom (135). Thus, further research on the ecology and natural history of this species alongside the pharmacokinetics of its venom is necessary in order to understand how procoagulant venom activity translates to a functional role for the animal.

**Figure 13:**
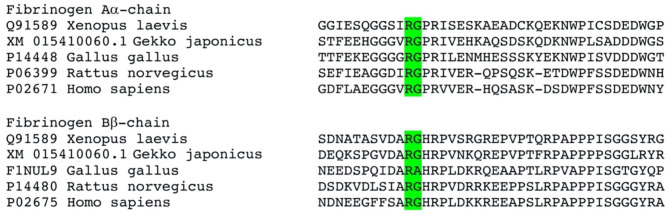
Sequence alignment of the activation cleavage sites. Cleavage site of normal thrombin is shown in green, however the venom induced cleavage sites remain to be elucidated for these venoms. There are however clear sequence differences downstream of the known thrombin cleavage site that are distinct between mammals and amphibians/diapsids.

The pseudo-procoagulant activity of the venoms also showed extreme taxon-specificity, being active on mammalian plasma but not amphibian plasma. The fibrinopeptide domain at which thrombin cleaves fibrinogen to form fibrin clots differs sharply in mammals vs. the homologous region of amphibians/diapsids (Figure 13). While the precise region at which the venoms cleave fibrinogen to form the unnatural fibrin clots has not been yet elucidated for these species, we observed a clear difference in clot strength between the thrombin-activated fibrinogen and that of the venom-activated fibrinogen (Figure 3). Much has been said in the literature about the inability of snake venom fibrionogenolytic enzymes to stabilize fibrin clots through the activation of Factor XIII, leading to weaker clots (136–142). However, this study (Figure 3) and previous research alike reported that venom-induced fibrin clots were still considerably weaker than thrombin-induced ones, even in the absence of FXIII, which is indicative of the venoms cleaving the fibrinogen differently relative to thrombin. Thus, snake venoms either cleave at a different region of the fibrinopeptide domain or at additional sites in the full-length fibrinogen chains to disrupt the latticework. Previous work on some species has revealed that some cleave only fibrinopeptide A, while others cleave only fibrinopeptide B, but with both at the same cleavage site as thrombin (143). However, cleaving at these sites should produce the same clot strengths as thrombin, yet they yield weaker clots. This suggests that if both fibrinopeptides are being cleaved at sites identical to those targeted by thrombin, yet produce weak, unstable, and short-lived clots, then the venoms are cleaving at additional sites, as would be the case for destructive (non-clotting) venoms. Such sites have been identified for some venoms (143, 144). Overall, however, this aspect of venom biochemistry is poorly researched. Future work should investigate whether the pseudo-procoagulant activity is mammal-specific by testing additional venoms on non-mammalian plasma. In addition, it is recommended to investigate the specific cleavage site to ascertain the differential nature of the cleavage between thrombin and the venoms.

### Clinical Implications

As previously discussed, procoagulant activation of zymogens would rapidly incapacitate prey animals *via* thrombosis. Conversely, the venom is diluted into a much larger blood volume in human bite victims, which typically does not result in stroke, although this has been noted on occasion (145, 146). Instead, when venom is diluted throughout a large blood volume, venom-induced consumption coagulopathy (VICC) occurs *via* depletion of clotting factors following excessive activity of the coagulation cascade (147, 148). This net anticoagulant state can result in death *via* internal bleeding.

Our findings demonstrate that the FX-activating procoagulant action of neonate *C. culminatus* venom is not neutralized by Antivipmyn®, one of the most frequently used antivenom products in Mexico. A logical explanation is that Antivipmyn® does not include *C. culminatus* venom in its immunizing mixture, relying on venom from adult *C. simus* specimens instead (112). *C. simus* has been recently split into *C. ehecatl* and *C. mictlantecuhtli* throughout most of its Mexican range (26), which is likely to affect antivenom manufacturing in turn. To our knowledge, no snake antivenom is produced using venom from juvenile individuals, and this is due to practical constraints of lower venom yields from smaller snakes. Venom from this species complex lacks the metalloprotease-driven true procoagulant trait, being instead pseudo-procoagulant *via* kallikrein-type serine proteases as shown in this work and previous studies (32, 44, 149). The clinical effects of this toxic activity would be VICC *via* depletion of fibrinogen following formation of unstable fibrin clots by serine proteases, as reported for multiple other species (51, 52). This SVSP-based pseudo-procoagulant activity was drastically reduced by Antivipmyn® in our assay for all venoms possessing this activity (and by AEBSF as well in the case of *C. mictlantecuhtli*). Our results therefore confirm extensive cross-reactivity for Antivipmyn® against pseudo-procoagulant SVSPs in contrast to the failure against the neonate *C. culminatus* Factor X activation.

The BIRMEX® (Faboterápico polivalente antiviperino precio), antivenom, widely marketed in Mexico alongside Antivipmyn® to treat rattlesnake envenoming, has *C. basiliscus* and *Bothrops asper* as its main immunizing species (112, 150). Although this product displays a high degree of cross-reactivity across multiple rattlesnake species (32, 150), further testing is recommended to determine whether it is able to neutralize the true procoagulant activity found in *C. culminatus*. However, as the immunizing venom composition does not include this species, it is unlikely to produce a more promising result.

Our findings draw attention to the pivotal importance of which immunizing venoms are chosen for antivenom production, including the critical need to ascertain ontogenetic changes. Such antivenom issues have been noted for other genera such as *Pseudonaja* (Australian brown snakes), with juvenile venoms rich in neurotoxic three-finger peptides to specialize on lizard prey, and adult venom rich in the FactorXa:FactorVa toxin complex to prey upon mammals as well at later life stages (58, 59, 110, 151). Thus, the antivenom raised against adults performs poorly against neonates due to the pronounced differences in venom biochemistry.

Unlike antivenom, the commercially available metalloprotease inhibitor Prinomastat was able to suppress the procoagulant action of neonate *C. culminatus* venom. However, the inhibitor DMPS performed poorly compared to Prinomastat, indicating that Prinomastat has greater potential as a field-deployable, temperature-stable, first-aid measure. Further problematic for DMPS is its intrinsic anticoagulant action upon plasma (elevating spontaneous clotting times) that might exacerbate disruption of blood coagulation in a real-life VICC scenario. Such a marked divergence may be ascribed to the different action of the two molecules. DMPS is a metal chelator, commonly used to treat heavy metal poisoning (152, 153), that binds the Zn^2+^ ions required for SVMPs to function (106). In contrast, peptidomimetic hydroxamate-based inhibitors like Prinomastat directly inactivate the activity of metalloproteases by binding to their catalytic site in combination with chelation of Zn^2+^ (105, 154). Thus, it is possible that DMPS is slower acting and/or requires a higher concentration to effectively hamper SVMP activity compared to Prinomastat. This was corroborated by preliminary tests showing that DMPS at a 20 mM concentration incubated with venom for 20 min was more efficient in neutralizing venom effects than at 2 mM for 2 min. Such a prolonged venom-inhibitor proximity would be unlikely to occur in a dynamic system like the bloodstream. Thus, investigations into the efficacy of inhibitors should prioritize those which are fast acting. It should also be noted that SVMPs are a highly diverse toxin family consisting of three classes, each of which is characterized by different structures and active domains with important consequences for their toxic activity (132). Thus, it is possible that the metalloproteases found in *Echis* venom, which were shown to be neutralized by DMPS (106) and by ion chelators in general better than by peptidomimetic inhibitors (105) differ from those present in *C. culminatus* to an extent where cross-reactivity is poor for DMPS. However, the study that examined the suitability of DMPS for neutralizing *Echis* venoms (106) used different methodologies (e.g., a kinetic fluorogenic assay to assess the effect of DMPS and other chelators on plasma clotting and SVMP activity) and thus comparing the relative potency with the poor neutralization results obtained in this study is impossible. Future work should undertake head-to-head comparisons between Prinomastat and DMPS using the presently used methodology and with a larger species pool (including *Echis*) to ascertain if DMPS consistently performs less efficiently than Prinomastat. However, in another study DMPS was conspicuously unable to neutralize *Daboia russelii* venom (155), which exerts its powerfully procoagulant effect *via* SVMP-induced Factor X activation like the neonate *C. culminatus* venom in this study. This suggests two future hypotheses to test. First, that the SVMPs in the two venoms, and thus presumably the FX activators in other viper venoms such as *Bothrops*, share a common molecular ancestry, putatively all being P-IIId SVMPs, whereby two lectin peptides are covalently linked to the SVMP enzyme. This would in turn suggest that DMPS is unable to neutralize P-IIId SVMPs in general. The prior work on DMPS examined only *E. carinatus* and *E. ocellatus*, which are both PIIIa rich venoms but not P-IIId rich like *E. coloratus* and putatively *E. leucogaster* and *E. pyramidum leakeyi* (56). Thus, future work should test a broader diversity of *Echis* to determine the efficacy of DMPS in neutralizing venoms rich in P-IIId SVMPs.

In recent years, several studies have proposed the use of small molecule inhibitors as an adjunct treatment for snakebite envenoming, to be administered before or alongside antivenom (105, 106, 156, 157). Both Prinomastat and DMPS are already licensed and widely marketed worldwide and can be administered outside a hospital setting (even *via* oral ingestion for DMPS), facilitating their use in real-life envenoming situations. Taken together, our observations indicate that *C. culminatus* possesses a peculiar venom phenotype that hampers antivenom cross-reactivity with its closest relatives in Mexico and encourages the use of metalloprotease inhibitors as an adjunct treatment. However, it should be noted that, while small molecule inhibitors have shown considerable potential in countering symptoms of snakebite, their repurposing for use as an adjunct treatment for envenomation will require *in vivo* investigations and clinical trials before regulatory authority approval.

From an epidemiological perspective, *C. culminatus* envenoming in humans is likely to occur regularly in the rural environments but is seldom documented due to poor epidemiology being a broad medical issue in such remote communities (Rebolledo, personal communication, January 2020), whereas *C. simus* (i.e., *C. mictlantecuhtli* + *C. ehecatl* + *C. simus*) is responsible for the majority of rattlesnake bite episodes in several Mexican states and Central American countries (14, 158). As populations centers spread into the remote areas occupied by *C. culminatus*, envenomations may increase in frequency. In addition, this species is sought after in the exotic pet trade and thus bites from captive *C. culminatus* individuals in the private reptile keeping sector may result in significant medical complications not neutralizable by available antivenoms, especially in countries where the species is not native. This species is therefore of potential clinical concern and we recommend further research on optimal treatments for its envenoming.

### Conclusion

This study reports the first occurrence of true procoagulant venom activity in Mexican Neotropical rattlesnakes for the species *Crotalus culminatus*, especially in early life stages. This went largely undetected in previous studies due to the lack of Ca^2+^ and phospholipids in plasma clotting assays resulting in experimental conditions lacking physiological venom requirements for functional activity. The poor efficiency of one of the main Mexican antivenom products against this action highlights the need to include a wide array of snake species and life-stages in antivenom immunizing mixtures. The metalloprotease inhibitor Prinomastat however was highly effective in neutralizing the procoagulant venom activity in *C. culminatus*, further validating the use of small molecule inhibitors as adjunct treatment for snakebite despite DMPS performing poorly in comparison. Overall, we hope our results will contribute to the evidence-based design of clinical management strategies for rattlesnake envenoming in Mexico and emphasize the importance of natural history and evolutionary research on rattlesnakes and their venom.

## Data Availability Statement

The raw data supporting the conclusions of this article will be made available by the authors, without undue reservation.

## Ethics Statement

The studies involving human participants were reviewed and approved by Human plasma was sourced from surplus supplies from the Australian Red Cross under Human Ethics Approval #2016000256. Written informed consent for participation was not required for this study in accordance with the national legislation and the institutional requirements. The animal study was reviewed and approved by Rhinella marina plasma was obtained under the University of Queensland Animal Ethics approval SBS/019/14/ARC.

## Author Contributions

LS, CZ, AC, and CR performed the experiments. LS, AC, and CR analyzed the data. EN-C, MB-V, and AA provided the venoms. BF designed the study. LS, CZ, and BF wrote the manuscript. All authors proof-read and revised the manuscript prior to submission.

## Conflict of Interest

The authors declare that the research was conducted in the absence of any commercial or financial relationships that could be construed as a potential conflict of interest.
